# Expert knowledge-based weighted *K*-means clustering for polycystic ovary syndrome phenotype identification

**DOI:** 10.3389/fendo.2026.1871986

**Published:** 2026-09-30

**Authors:** Priyanka D., Sumangali K.

**Affiliations:** School of Computer Science Engineering and Information Systems, Vellore Institute of Technology, Vellore, India

**Keywords:** biomarker analysis, expert knowledge integration, false discovery rate (FDR), K-means clustering, mutual information, PCOS, phenotyping, silhouette score

## Abstract

**Background:**

Polycystic ovary syndrome (PCOS) is a heterogeneous endocrine disease condition that affects 8%–13% of the female population worldwide. Its clinical heterogeneity makes the binary approach to phenotyping less effective for the biological significance of such phenotypic groups and the personalized management of PCOS. To overcome this issue, we present the expert knowledge-based weighted *K*-means (EKWKM) approach.

**Objective:**

The proposed EKWKM is a new, weakly supervised phenotyping approach that uses structured clinical knowledge along with mutual information (MI)-based feature weighting, whereby the feature weighting process is weakly supervised, but clustering is unsupervised.

**Methods:**

The EKWKM was benchmarked using a clinical dataset (*n* = 541, 41 features) and a large-scale population study (*n* = 120,000). A grid search was performed for *K* ∈ {2, 3, 4} and *α* = 0.1–0.9. The silhouette score (95% bootstrap confidence interval), the adjusted Rand index (ARI), and the Davies–Bouldin (DB) index were used to quantify cluster quality. Biomarker importance was measured using the Kruskal–Wallis test with Benjamini–Hochberg correction and *ϵ*^2^ effect size.

**Results:**

The most effective clinical model (*K* = 2, *α* = 0.9) achieved a silhouette score of 0.3606. The model also achieved ARI = 0.9780 ± 0.0362 and DB = 1.1480 and identified 13 significant biomarkers. The population survey acted as a negative control dataset [random forest area under the curve (AUC) = 0.4974 ± 0.0096] and demonstrated no phenotypic signal. Cross-dataset transfer decreased Cramér’s *V* from 0.497 to 0.006, which confirmed that EKWKM did not add false patterns. The main part of the clustering was due to data-driven MI, as confirmed by the ablation analysis. Expert knowledge improved clustering robustness.

**Conclusion:**

EKWKM formed clinically meaningful and Rotterdam-based PCOS phenotype clusters. In contrast, the population survey clusters formed symptom-reporting patterns and are not meaningful PCOS phenotypes.

## 1 Introduction

Among women of reproductive age, polycystic ovary syndrome (PCOS) is one of the most common endocrine conditions, with global prevalence estimates ranging from 8% to 13% when the Rotterdam criteria are applied, rising to approximately 21% under broader diagnostic frameworks (1, 2). The syndrome presents heterogeneously encompassing excess androgen levels, dysfunction of the ovary, polycystic ovarian morphology, insulin resistance, dyslipidemia, and type II diabetes mellitus risk in an elevated manner (3). A recent landmark investigation in Nature Medicine published by Gao et al. (4) identified four data-driven subtypes characterized by hyperandrogenism, obesity, high sex hormone-binding globulin (SHBG), and high luteinizing hormone (LH) and anti-Müllerian hormone (AMH), across five international cohorts comprising 11,908 women. Much clinical variability of PCOS remains a challenge, which has complicated its diagnosis and treatment planning. Four phenotypes are recognized by the Rotterdam consensus; however, this model only describes the biological diversity experienced in clinical practice partially (5). Machine learning (ML)-driven clustering techniques are increasingly being used to identify statistically sound and clinically meaningful patient subgroups (6–8). The versatility of the clustering algorithms across heterogeneous domains has been demonstrated from patient stratification in oncology to population segmentation under public health surveillance (24). Nevertheless, to be directly applied to clinical data, the domain-specific improvements need to be interpretable for relevant diagnosing (26). Multicenter analysis showed that hierarchical clustering of hormonal profiles yields reproducible subgroups with unique genetic architecture, providing independent biological support for data-driven phenotyping methodologies (8). *K*-means clustering has been popular in patient stratification; however, the standard version of the algorithm assumes equal properties among all features, which fails to prioritize diagnostically relevant specific markers. Mutual information (MI) provides a systematic model of nonlinear feature–outcome interactions, although is less reliable in small or noisy clinical data (22). In PCOS, a recent systematic review established a 29% clustering-based account of artificial intelligence (AI)/ML studies, which is a significant gap in phenotype discovery (7). In a bid to overcome these problems, the research in this paper suggests the expert knowledge-based weighted *K*-means (EKWKM) framework. The key contributions are (i) a hybrid expert -mutual information feature-weighting algorithm; (ii) dual-dataset validation on a small clinical cohort (*n* = 541) and a large population dataset (*n* = 120,000); and (iii) an ablation study isolating the contribution of each weighting component, together with a false discovery rate (FDR)-corrected biomarker analysis with 95% bootstrap confidence intervals.

## 2 Related work

### 2.1 PCOS phenotyping based on machine learning

As reported in a systematic review conducted by Barrera et al. (6), ML and AI methods were applied for the classification of PCOS, with hierarchical clustering methods generating metabolic and reproductive sub-phenotypes. Dapas et al. (9), who analyzed PCOS in a multi-ethnic cohort of >2,000 individuals, applied the non-negative matrix factorization method, proving the presence of subtypes determined by insulin resistance and androgen excess. Gao et al. (4) published an influential article in Nature Medicine, in which they identified four data-driven PCOS subtypes among 11,908 women. Van der Ham et al. (8) identified three strong subtypes based on hierarchical clustering with genetic associations. Wang et al. (12) showed an AI-driven approach that identified the existence of insulin-resistant phenotypes in PCOS and distinguished PCOS subgroups that can be managed effectively. Aggarwal and Pandey (23) showed that ML-based stratification of PCOS reveals variations not captured by traditional criteria such as the Rotterdam criteria. From a methodological perspective, Emara et al. (28) emphasized that the phenotypes obtained through ML significantly increase the diagnostic accuracy, but relatively lack attention to unsupervised learning approaches. Apart from methodological innovations, the assessment of clustering quality has become a vital issue. Previous studies have revealed that high-level validation methods, such as Cohen’s kappa and the *F*-score, play a crucial role in determining the level of consistency. Currently, consensus clustering has become complicated for heterogeneous data (10).

### 2.2 Feature weighting in clustering

Huang et al. (13) suggested that the refinement of the feature weights should be performed using the algorithm of weighted *K*-means, which is based on the within-cluster dispersion in an iterative way. Joshi (29) augmented the clinician-assigned importances with data-driven weights on PCOS subtype stratification and showed better interpretability and phenotype accuracy. AI-Khamees et al. (25) demonstrated that, when compared with other distance and cluster refinement-based feature weighting methods, hybrid distance and cluster refinement-based feature weighting methods have a better silhouette score in the clustering of medical datasets. Osman (30) used clustering of medical datasets and integrated *K*-means for strong performance in cardiovascular disease classification. Gu et al. (14) formalized the data- and expert-driven reasoning complement in medical AI through a domain/expert-guided hybrid framework.

### 2.3 Research gap

Nevertheless, no previous study has: i) exhaustively searched the *α* mixing parameter using grid search; ii) demonstrated a single hybrid framework on a small clinical cohort and a large population sample simultaneously; and iii) empirically determined the contribution of each weighting term in an empirically grounded ablation study. The EKWKM framework helps to address all three weaknesses.

## 3 Materials and methods

### 3.1 Datasets

Two PCOS datasets were used. Dataset 1 is a publicly available, clinically curated dataset from the Kaggle (*n* = 541, 41 clinical features) cohort of patients with PCOS. The second dataset, a large population survey (*n* = 120,000), is also publicly available from Kaggle. The links for both datasets are available in the “*Data availability statement*.” A summary of the analysis of their differences is shown in **Table 1**. The overall EKWKM workflow is shown in **Figure 1**.

**Table 1:** Analysis of the differences in the characteristics of dataset 1 (targeted clinical PCOS cohort, *n* = 541) and dataset 2 (large-scale population survey, *n* = 120,000).

Property	Dataset 1: clinical (*n* = 541)	Dataset 2: population (*n* = 120,000)
Source	PCOS_DATASET_PRASOON.csv	pcos_prediction_dataset.csv
Records	541 patients	120,000 records
Features	41 clinical features	15 population survey features
PCOS-positive	177 (32.7%)	12,595 (10.5%) diagnosis+
Top MI features	Follicle number (R/L), skin pigmentation changes, increased hair growth, weight gain	Awareness of PCOS, fertility concerns, menstrual regularity, hirsutism, urban/rural
Expert weight source	Panel of 4 experts	Panel of 4 experts
Optimal (*K*, *α*)	*K* = 2, *α* = 0.9	*K* = 4, *α* = 0.1

PCOS, polycystic ovary syndrome; MI, mutual information; R, right; L, left.

**Figure 1:**
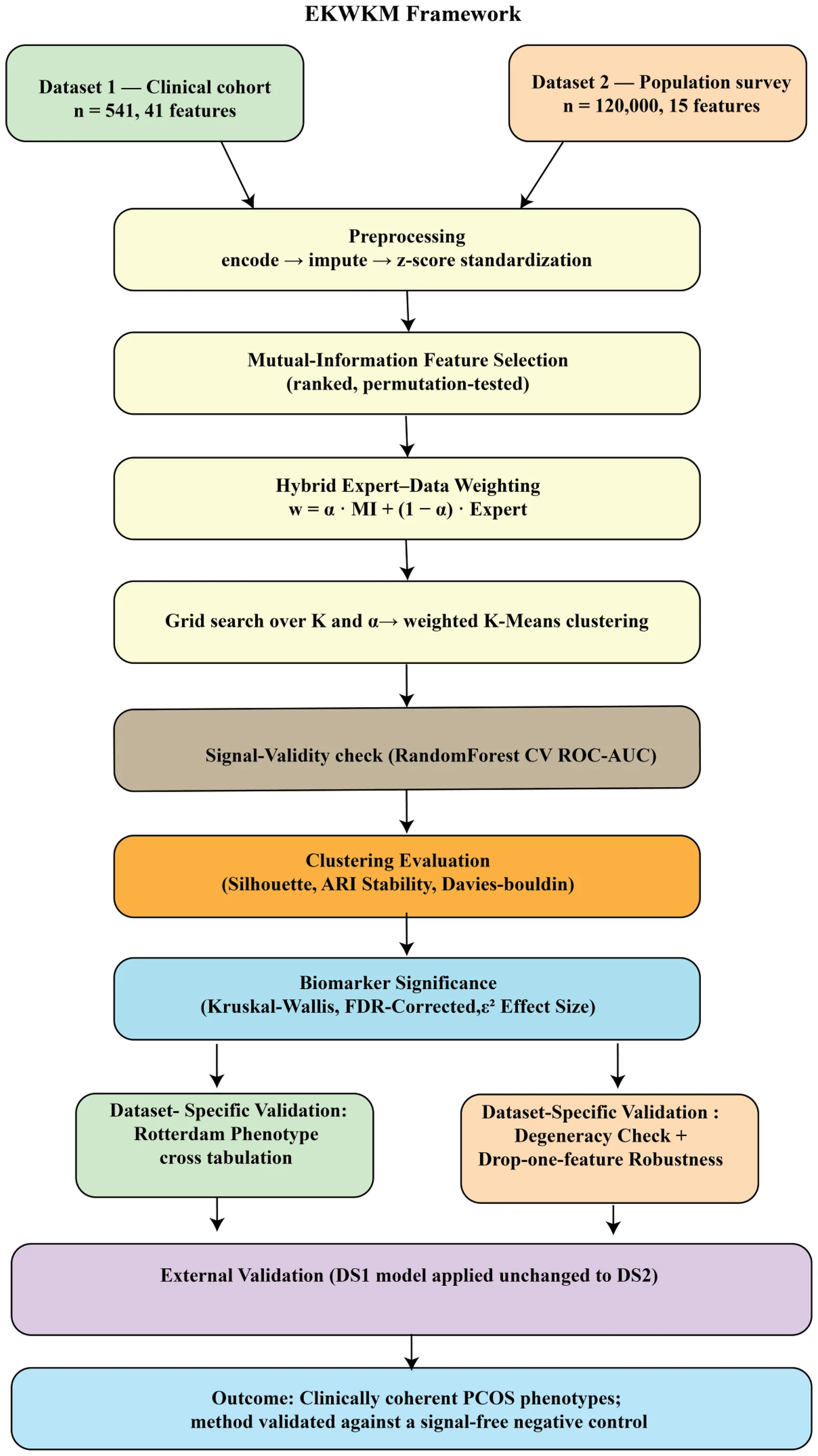
Flowchart of the proposed expert knowledge-based weighted *K*-means (EKWKM) framework. An identical pipeline pre-processing, mutual information computation, hybrid expert–data weighting, and a grid search over *K* and alpha was used for both datasets. The two branches only split at the final validation step, which had to be different for each dataset as only dataset 1 included the clinical details (e.g., follicle counts) needed to apply Rotterdam phenotyping.

### 3.2 Data pre-processing

Each dataset was consistently pre-processed. To avoid data leakage issues, administratively relevant pieces of information such as the sl.no and patients’ ID were removed. The categorical variables were coded using integers. The column-median technique was used to impute missing values as this method is insensitive to skewness and outliers on a random basis. There were no issues with missing data in either dataset. For dataset 1, there were only two missing observations (one missing observation per variable among the total number of observations, which was 0.18%). For dataset 2, “acne severity” had 60,085 missing observations (out of 120,000 total observations, 50.07%), while the remaining variables had no missing observations. This variable was kept with missingness coded as a separate category instead of being imputed due to the categorical nature of the variable. Sensitivity analysis showed that this did not affect the reported results as acne severity was ninth out of the 15 most informative variables and did not reach the top 5 feature list. The same pre-processing method was applied to validate both datasets, which would not change the results as dataset 2 does not have any missing values; thus, mean or median imputation would make no difference for this dataset. Two hormone levels were measured in dataset 1 [i.e., AMH and beta human chorionic gonadotropin (β-HCG)], which were calculated as string variables. A sensitivity analysis in which these were treated as numeric values revealed the results to be essentially the same [agreement adjusted Rand index (ARI) = 0.985], with an improved internal structure; hence, the findings were robust to such an alternative treatment of these variables. All variables were scaled using the *z*-score method (mean = 0, SD = 1). However, in the case of dataset 2, imputation of the missing data using the median was performed before scaling due to several missing data points.

### 3.3 Mutual information

MI was calculated between the individual features and the diagnostic label with the mutual_info_classif in scikit-learn with random state = 42 to be reproducible (22). The MI score was defined as:


$$\mathrm{MI}(R;S)=\sum_{r}\sum_{s}p(r,s)\cdot \log[\frac{p(r,s)}{p(r)p(s)}]$$

The joint probability is expressed as *p*(*r*,*s*), whereas the marginal probabilities are expressed as *p*(*r*) and *p*(*s*). Feature selection selected the highest-ranking features in terms of MI (20 highest-ranking features from dataset 1 and five from dataset 2). For the theoretical background of this approach, rather than fixing an arbitrary cutoff point, this estimation showed how many features had non-random information using a permutation test. MI was calculated according to 200 permutations of the class label, and the feature was defined as an informative feature if it had MI above the 95th percentile of the null distribution after Benjamini–Hochberg (BH) correction. In this case, dataset 1 had 11 informative features (all follicle count, hyperandrogenism, cycle, and key hormones) among 41, capturing 77% of the MI; thus, the cutoff of 20 highest-ranking features for clustering is a conservative choice of informative features. There were no informative features in dataset 2, which is expected in a negative control (see *Section 5.1*). Feature selection is a critical step in predicting disease risk using ML.

### 3.4 Hybrid expert–data weighting

For each selected feature *f*, the hybrid weight is computed as a convex combination:


$$w(f,\alpha )=\alpha \cdot {\text{MI}}_{\text{norm}}(f)+(1-\alpha )\cdot {\text{Expert}}_{\text{norm}}(f)$$

where *α* ∈ {0.1, …, 0.9} controls the balance between the data-driven (*α*→1) and the expert-driven (*α*→0) components.

Because the MI measure is based on the diagnostic variable (PCOS Y/N in dataset 1 and diagnosis in dataset 2), the weighting of the feature process utilizes weakly supervised learning, not unsupervised, as it uses the information present in the class labels to compute the MI measure, and only the following clustering stage is an unsupervised learning algorithm. This is a conscious decision since embedding the diagnostic information into the weights of the features is how the medical knowledge and the signal from the data interact. However, this results in a self-referential system, whereby the phenotypes discovered are influenced by the same labels that will validate them (*Section 5.4*).

The MI scores and expert weights were then min–max normalized in the range [0,1] and aggregated. For both datasets 1 and 2, the expert weights were obtained from a panel of four experts who had prior experience in the diagnosis and treatment of patients with PCOS (the panel consisted of two gynecology/obstetrics experts and two doctors from a tertiary and government hospital, with individual details available in the **Supplementary Material**). Each expert individually scored each candidate feature on a 0–10 scale for its diagnostic importance in PCOS. The sum of the scores from all four experts (maximum possible sum = 40) was min–max normalized in the range [0,1] to obtain the expert weight for a particular feature. This was done independently by the experts and not via the Delphi consensus, an issue pointed out in *Section 5.4*. $X_{w}$ = X ⊙ *w*(*f*, α). This is directly input into *K*-means, with $X_{w}$ being the weighted feature matrix.

### 3.5 Clustering and search of optimal configurations

The grid search was done on *K* ∈ {2,3,4} in dataset 1 and on *K* ∈ {2,3,4} in dataset 2 and *α* ∈ {0.1 …, 0.9}. For the initializations, each *K*-means execution ran 100–200 random initializations with constant random state = 42. Configuration clusters of less than 10 members were dropped. Maximizing the silhouette score was used to select the best configuration with ARI = 0.85 (or 0.88 in the case of dataset 1).

### 3.6 Cluster validation metrics

The quality and the stability of the clusters were measured using three different metrics: two metrics for internal validity, i.e., the silhouette score and the Davies–Bouldin (DB) index, and a third metric for subsampling stability, the ARI. The silhouette score (15) measures the intra-cluster cohesion *versus* the inter-cluster separation (on a scale from −1 to +1, with high values being desirable) using 95% bootstrap confidence intervals (based on 1,000 iterations). The DB index (16) computes the ratio of within-cluster dispersion over the between-cluster separation (low values are desirable). The ARI (17) in this study was not used in its classical sense as the agreement of the clustering with the external reference labeling, but rather as the subsampling stability metric: the clustering of the whole dataset was compared with the clustering of the subsamples (20–30 cycles using 80%–90% of the data). Therefore, ARI ≈ 1.0 means the consistency of the partition across different subsamples and not the agreement with the ground-truth labels. All ARI calculations were conducted using a fixed random seed (random_state = 42). The silhouette score and the DB index are two internal validation techniques widely suggested for clustering analysis (27), and the use of multiple validity techniques is encouraged by recent benchmarking works (11).

### 3.7 Biomarker statistical analysis

The Kruskal–Wallis *H* test (18) is a non-parametric test that is used to assess the differential expression of each biomarker within clusters as opposed to one-way ANOVA. BH-FDR correction was applied to control multiple comparisons (19). The magnitude of the effect was evaluated using the epsilon squared (*ε*^2^) statistic for the Kruskal–Wallis test.


$${\text{ϵ}}^{2}=max(0,(H-k+1)/(n-k))$$

where *H* is the Kruskal–Wallis statistics, *k* is the number of clusters, and *n* is the total number of observations. Effects were considered as small (*ε*^2^ < 0.06), moderate (0.06 ≤ *ε*^2^ < 0.14), or large (*ε*^2^ ≥ 0.14) based on small/moderate/large effects bands, respectively (20). It should be noted that such thresholds are applied to *ε*^2^ here for interpretational purposes and not as specific measurement cutoffs.

## 4 Results

### 4.1 Optimum cluster structure (*K*) and mixing coefficient (*α*)

The results obtained from the grid search on the two datasets are presented in **Table 2**. For the clinical dataset (*n* = 541), *K* = 2 had the highest silhouette in the searched grid at *α* = 0.9 (silhouette = 0.3606, 95%CI = 0.3381–0.3866, ARI = 0.9780 ± 0.0362, DB = 1.1480). *K* = 3 and *K* = 4 were not rejected for every α; valid, non-degenerate configurations existed at α = 0.8 -0.9 for *K* = 3 and at α = 0.9 for *K* = 4, but none exceeded the silhouette of *K* = 2; therefore, *K* = 2 was selected. For the negative control analysis of dataset 2 (*n* = 120,000) (silhouette = 0.5023, ARI = 0.9654 ± 0.0891, DB = 0.8867), *K* = 4 and *α* = 0.1 were kept for configuration as the reference configuration instead of the higher-silhouette configurations at larger *α*. The reasons for this are discussed in Sections 4.3.1 and 5.1.

**Table 2:** Grid search results over *α* ∈ {0.1 …, 0.9} for both datasets for *K* ∈ {2,3,4}.

*K*	*α*	Silhouette score	ARI (mean ± SD)	Davies–Bouldin	FDR sig.	Status
Dataset 1: clinical PCOS (*n* = 541)						
2	0.1	0.1966	0.7194 ± 0.4354	2.0495	13/20	Low ARI
2	0.2	0.2036	0.9443 ± 0.0399	2.0074	13/20	Valid
2	0.3	0.2107	0.9397 ± 0.0319	1.9542	13/20	Valid
2	0.4	0.2243	0.9551 ± 0.0506	1.8614	13/20	Valid
2	0.5	0.2397	0.9618 ± 0.0334	1.7567	14/20	Valid
2	0.6	0.2627	0.9580 ± 0.0309	1.6056	14/20	Valid
2	0.7	0.2850	0.9820 ± 0.0135	1.4674	14/20	Valid
2	0.8	0.3214	0.9442 ± 0.0350	1.3038	14/20	Valid
2★	0.9	0.3606 (0.3381–0.3866)	0.9780 ± 0.0362	1.1480	13/20	BEST
3	0.1–0.6	–	–	–	–	Rejected
3	0.7	0.1591	0.5374 ± 0.1890	1.9063	15/20	Low ARI
3	0.8	0.1813	0.9206 ± 0.0545	1.7631	12/20	Valid
3	0.9	0.2239	0.9438 ± 0.0568	1.4986	13/20	Valid
4	0.1–0.7	–	–	–	–	Rejected
4	0.8	0.2069	0.7637 ± 0.1082	1.5408	13/20	Low ARI
4	0.9	0.2260	0.9208 ± 0.0649	1.5306	14/20	Valid
Dataset 2: population survey PCOS (*n* = 120,000)						
2	0.1	0.3584	0.1002 ± 0.2999	1.4201	1/5	Low ARI
2	0.2	0.3471	0.6001 ± 0.4898	1.4410	1/5	Low ARI
2	0.3	0.3498	1.0000 ± 0.0000	1.4003	1/5	Degenerate
2	0.4	0.3499	1.0000 ± 0.0000	1.3756	1/5	Degenerate
2	0.5	0.3467	1.0000 ± 0.0000	1.3766	1/5	Degenerate
2	0.6	0.3399	1.0000 ± 0.0000	1.4162	1/5	Degenerate
2	0.7	0.4402	1.0000 ± 0.0000	1.0857	1/5	Degenerate
2	0.8	0.5572	1.0000 ± 0.0000	0.7946	1/5	Degenerate
2	0.9	0.6684	1.0000 ± 0.0000	0.5778	1/5	Degenerate
3	0.1	0.4356	1.0000 ± 0.0000	1.0733	2/5	Degenerate
3	0.2	0.4175	0.9729 ± 0.1180	1.1055	2/5	Valid
3	0.3	0.4050	1.0000 ± 0.0000	1.1227	2/5	Degenerate
3	0.4	0.3870	0.9503 ± 0.1466	1.1817	2/5	Valid
3	0.5	0.3367	0.6426 ± 0.1672	1.3399	2/5	Low ARI
3	0.6	0.3789	1.0000 ± 0.0000	1.1256	2/5	Degenerate
3	0.7	0.4614	1.0000 ± 0.0000	0.8977	2/5	Degenerate
3	0.8	0.5593	1.0000 ± 0.0000	0.7072	2/5	Degenerate
3	0.9	0.6674	1.0000 ± 0.0000	0.5121	2/5	Degenerate
4★	0.1	0.5023	0.9654 ± 0.0891	0.8867	3/5	Valid (chosen)
4	0.2	0.4743	0.9581 ± 0.0909	0.9143	3/5	Valid
4	0.3	0.4455	0.9773 ± 0.0540	0.9550	3/5	Valid
4	0.4	0.4081	0.8769 ± 0.1556	1.0265	3/5	Valid
4	0.5	0.3731	0.7962 ± 0.1827	1.0330	3/5	Low ARI
4	0.6	0.4613	1.0000 ± 0.0000	1.0810	2/5	Degenerate
4	0.7	0.5211	1.0000 ± 0.0000	0.9117	2/5	Degenerate
4	0.8	0.5878	1.0000 ± 0.0000	0.7505	2/5	Degenerate
4	0.9	0.6816	1.0000 ± 0.0000	0.5558	2/5	Degenerate

★ indicates the selected configuration. Values were computed with a fixed seed (random_state = 42) using subsampling ARI. For dataset 1, *K* = 3 and *K* = 4 yielded valid configurations only at α = 0.8 -0.9 and α = 0.9, respectively, but none exceeded the silhouette of *K* = 2. For dataset 2, *K* = 4, α = 0.1 was retained as the reference configuration instead of higher-silhouette settings that reach the discrete-space ARI ≈ 1.000 ceiling ("Degenerate"). "Low ARI" marks configurations with ARI < 0.85; configurations producing a cluster with fewer than 10 samples were excluded ("Rejected"). FDR, false discovery rate.

FDR, false discovery rate.

The picture was very different from that of the population dataset. The *K* = 2 setups did not reach the ARI threshold of 0.85 for the majority of the *α* values, which is logical as a binary partition is unlikely to reflect the spectrum of presentations in the case of 120,000 people. For the selected *K* = 4, *α* = 0.1 configuration, the silhouette measure was 0.5023 and the ARI was 0.9654 ± 0.0891 over 20 subsamples (DB = 0.8867). The silhouette was higher than that for the clinical population; however, this does not indicate quality. Internal measures tend to favor the geometric compactness of a small number of discrete points, which cannot be relied upon here. The grid contains the majority of other configurations with an invariant ARI of 1.0000 (**Table 2**). This is a consequence of the discreteness of the five binary survey features (32 total value combinations), which leads to perfect partitions in each subsample due to the categorical nature of the feature space. The ARI (0.9654 ± 0.0891) of the reference configuration was close to but below this ceiling and was preferred over the exactly degenerate alternatives for this reason (see *Section 6*).

### 4.2 Dataset 1: clinical PCOS results

#### 4.2.1 Clustering performance and phenotype structure

The best configuration (*K* = 2, *α* = 0.9) included 541 patients in cluster 0 (*N* = 180, 33.3%) and cluster 1 (*N* = 361, 66.7%), close to the PCOS+ actual frequency (32.7% is hence “face valid”). The silhouette score of 0.3606 was a 130.3% improvement from the actual computed (weighted) unweighted baseline (0.1565), and the ARI of 0.97800.0362 over 30 resamples suggests a robust partition. The alpha sensitivity (**Figure 2**) on dataset 1 (*K* = 2) showed a monotonic increase in the silhouette and DB scores toward 0.9 within the sampled grid. **Figure 3** shows the best-performing *K* comparisons with error bars. **Figure 4** displays the 2D projection on *t*-distributed stochastic neighbor embedding (*t*-SNE; perplexity = 30) showing distinct, well-separated clusters (0 and 1).

**Figure 2:**
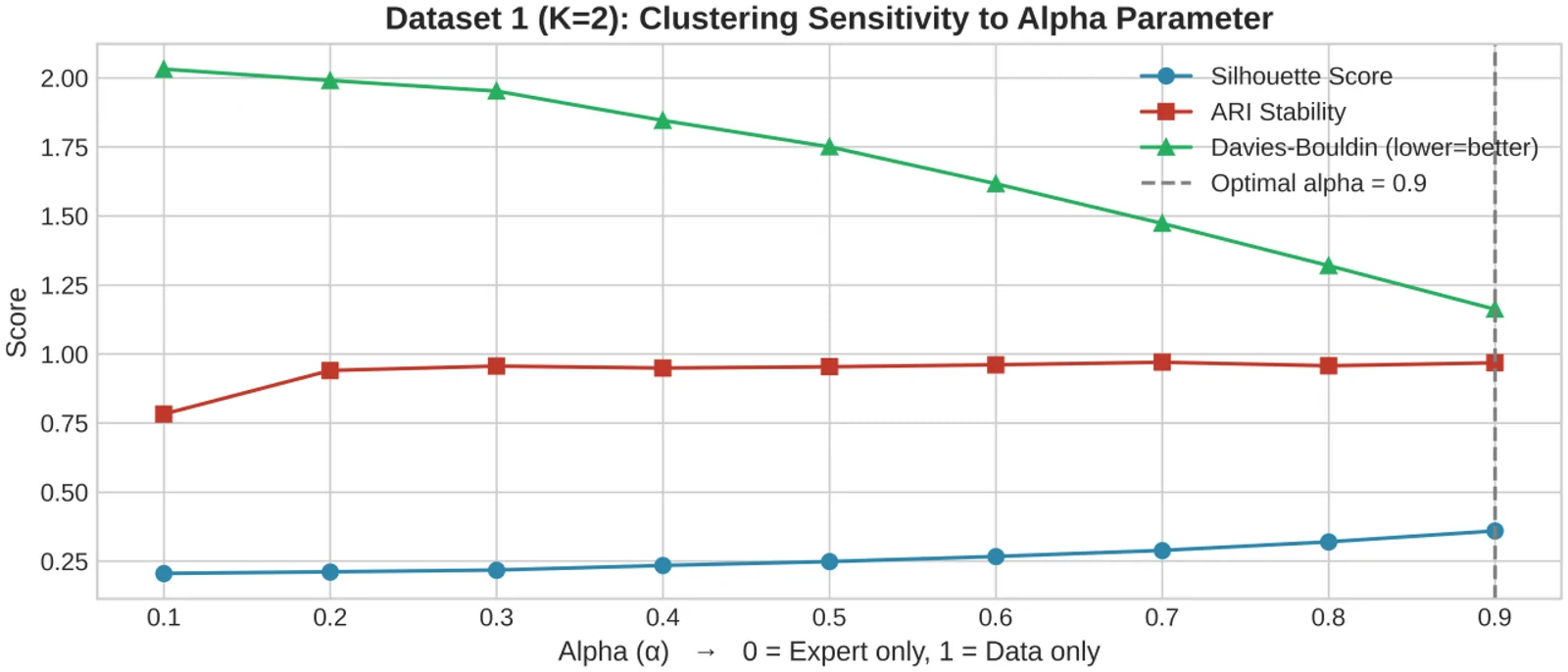
Alpha sensitivity analysis for dataset 1 (*K* = 2). The silhouette score and the adjusted Rand index (ARI) increase monotonically with *α*, while the Davies–Bouldin (DB) index decreases, confirming *α* = 0.9 as optimal.

**Figure 3:**
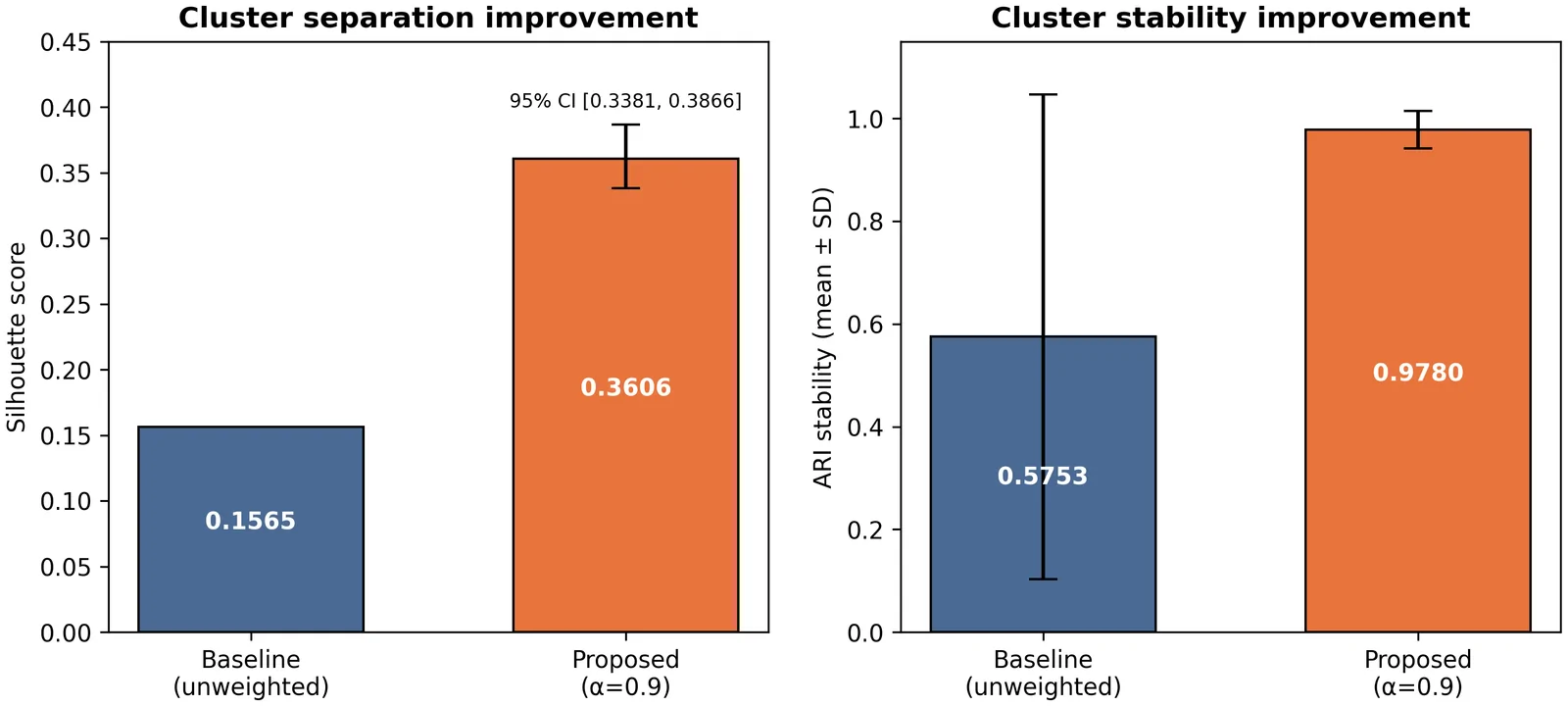
Performance comparison with 95% bootstrap confidence interval error bars for dataset 1 (*K* = 2, *α* = 0.9). Left, Silhouette score improvement over unweighted baseline. Right, Adjusted Rand index (ARI) stability improvement.

**Figure 4:**
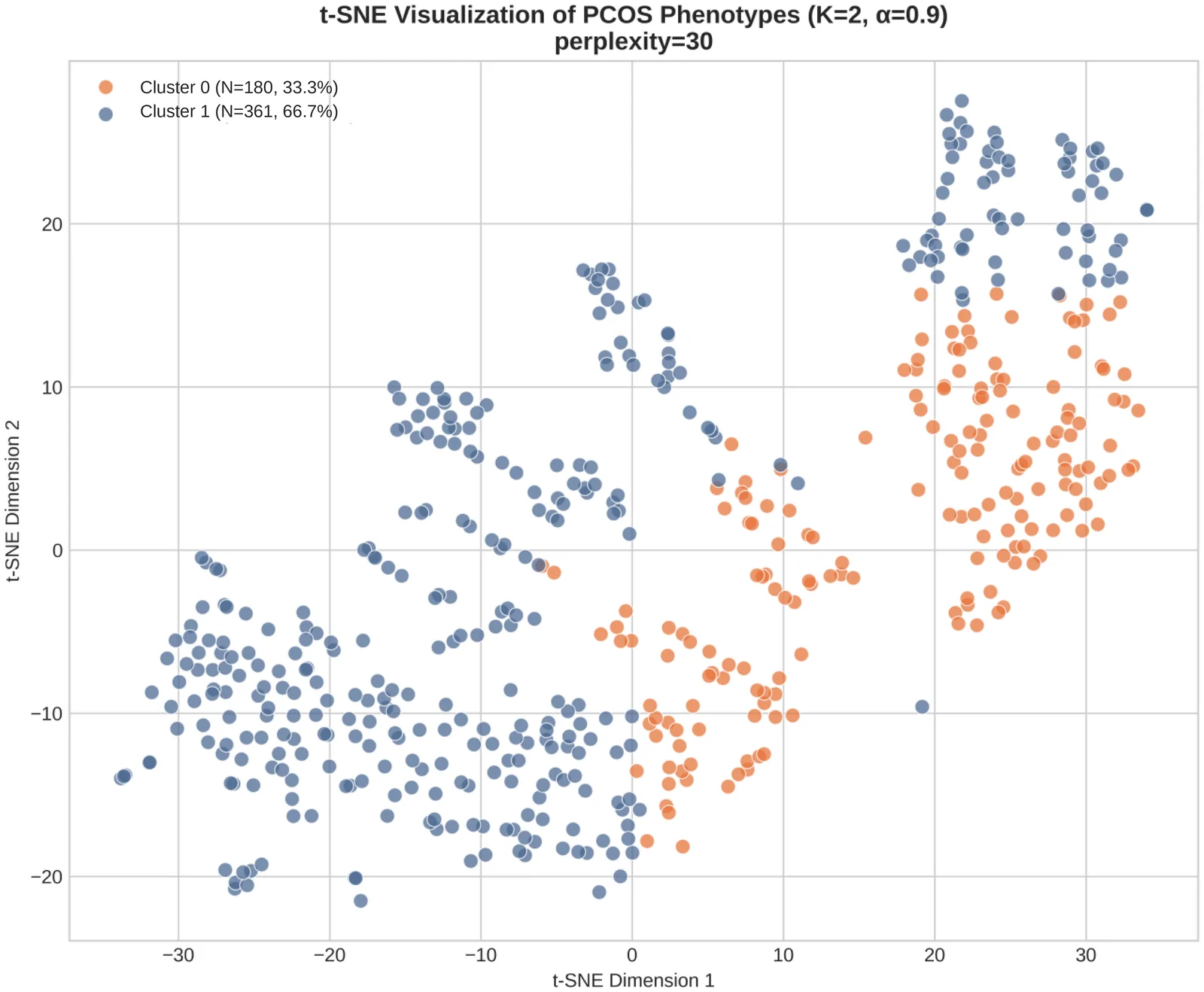
*t*-Distributed stochastic neighbor embedding (*t*-SNE) 2D projection of dataset 1 weighted feature space (*K* = 2, *α* = 0.9, perplexity = 30, n_iter = 1,000). Cluster 0 (*N* = 180, *red*) corresponds to the active polycystic ovary syndrome (PCOS) phenotype and cluster 1 (*N* = 361, *blue*) to mild/subclinical presentations.

#### 4.2.2 Biomarker significance: dataset 1

As additional (non-circular) control for the concern that the clustering features might necessarily be circular by definition, the analysis was rerun using only the 21 features that were not among the top 20 MI-selected features. Of the 21 held-out features, eight remained FDR-significant: BMI (*ε*^2^ = 0.032), waist circumference (*ε*^2^ = 0.029), hip circumference (*ε*^2^ = 0.028), left avg. follicle size (*ε*^2^ = 0.023), pulse rate (*ε*^2^ = 0.018), age (*ε*^2^ = 0.017), β-HCG I (*ε*^2^ = 0.009), and years married (*ε*^2^ = 0.009). All minor effects provided direct evidence that such features are non-circular evidence that the clusters relate to real physiology rather than being a mere reiteration of the features that build them.

Of the 20 features, 13 were statistically significant in the Kruskal–Wallis test using the BH-FDR correction for *q* < 0.05 (**Table 3**). Only two biomarkers had a large effect size (*ε*^2^ ≥ 0.14), and both appeared biologically plausible—the number of bilateral follicles on the right (*ε*^2^ = 0.6185) and left (*ε*^2^ = 0.5521)—which were used for the diagnosis of polycystic ovarian morphology, a characteristic feature of the condition. Another five features received a medium effect size (0.06 ≤ *ε*^2^ < 0.14): skin darkening (*ε*^2^ = 0.137, suggesting insulin resistance-associated acanthosis nigricans), excessive hair growth (*ε*^2^ = 0.121, related to hyperandrogenism), irregular cycle (*ε*^2^ = 0.086), weight gain (*ε*^2^ = 0.084), and fast food consumption (*ε*^2^ = 0.066). These results, where the algorithm picked up on these specific biological clues, only strengthened the premise about the underlying coherent biological subgroups in dataset 1. Simultaneously, this is a label-dependent selection based on the relative information contribution of each feature on disease labelling, and this limitation will be elaborated on in *Section 5*. The results are visualized in **Figures 5** and **6**, which show a heat map based on the selected biomarkers (*z*-scores in **Figure 5**) and box plots for the top three FDR-significant variables (**Figure 6**).

**Table 3:** Kruskal–Wallis test results with Benjamini–Hochberg false discovery rate (FDR) correction for dataset 1 (*K* = 2, *α* = 0.9).

S. no.	Biomarker	Raw *p*-value	FDR *q*-value	*H*-statistic	${\text{ϵ}}^{2}$	Effect size	Sig.
1	Follicle no. (R)	1.06 × 10^−74^	2.13 × 10^−73^	334.39	0.6185	Large	Yes
2	Follicle no. (L)	6.66 × 10^−67^	6.66 × 10^−66^	298.60	0.5521	Large	Yes
3	Skin darkening	5.07 × 10^−18^	3.38 × 10^−17^	74.85	0.1370	Medium	Yes
4	Hair growth (Y/N)	4.29 × 10^−16^	2.14 × 10^−15^	66.10	0.1208	Medium	Yes
5	Cycle (R/I)	5.83 × 10^−12^	2.33 × 10^−11^	47.38	0.0861	Medium	Yes
6	Weight gain (Y/N)	1.08 × 10^−11^	3.61 × 10^−11^	46.17	0.0838	Medium	Yes
7	Fast food (Y/N)	1.44 × 10^−09^	4.11 × 10^−09^	36.61	0.0661	Medium	Yes
8	Cycle length (days)	1.67 × 10^−06^	4.17 × 10^−06^	22.94	0.0407	Small	Yes
9	Weight (kg)	6.40 × 10^−06^	1.42 × 10^−05^	20.36	0.0359	Small	Yes
10	Pimples (Y/N)	3.14 × 10^−05^	6.28 × 10^−05^	17.33	0.0303	Small	Yes
11	Avg. *F* size (R) (mm)	6.18 × 10^−3^	1.12 × 10^−2^	7.50	0.0121	Small	Yes
12	AMH (ng/ml)	8.42 × 10^−3^	1.40 × 10^−2^	6.94	0.0110	Small	Yes
13	FSH (mIU/ml)	3.01 × 10^−2^	4.63 × 10^−2^	4.70	0.0069	Small	Yes
–	RR, FSH/LH, PRL, TSH, vit. D3, LH, waist/hip	>0.05	>0.05	<4.2	~0.000–0.006	NS	No

Effect sizes: small (*ε*^2^ < 0.06), medium (0.06–0.14), and large (≥0.14).

NS, not significant; R/I, regular/irregular (cycle); R/L, right/left (ovary); N, no; AMH, anti-Müllerian hormone; FSH, follicle-stimulating hormone; prolactin; TSH, thyroid-stimulating hormone; PRL, prolactin; TSH, thyroid-stimulating hormone; LH, luteinizing hormone.

**Figure 5:**
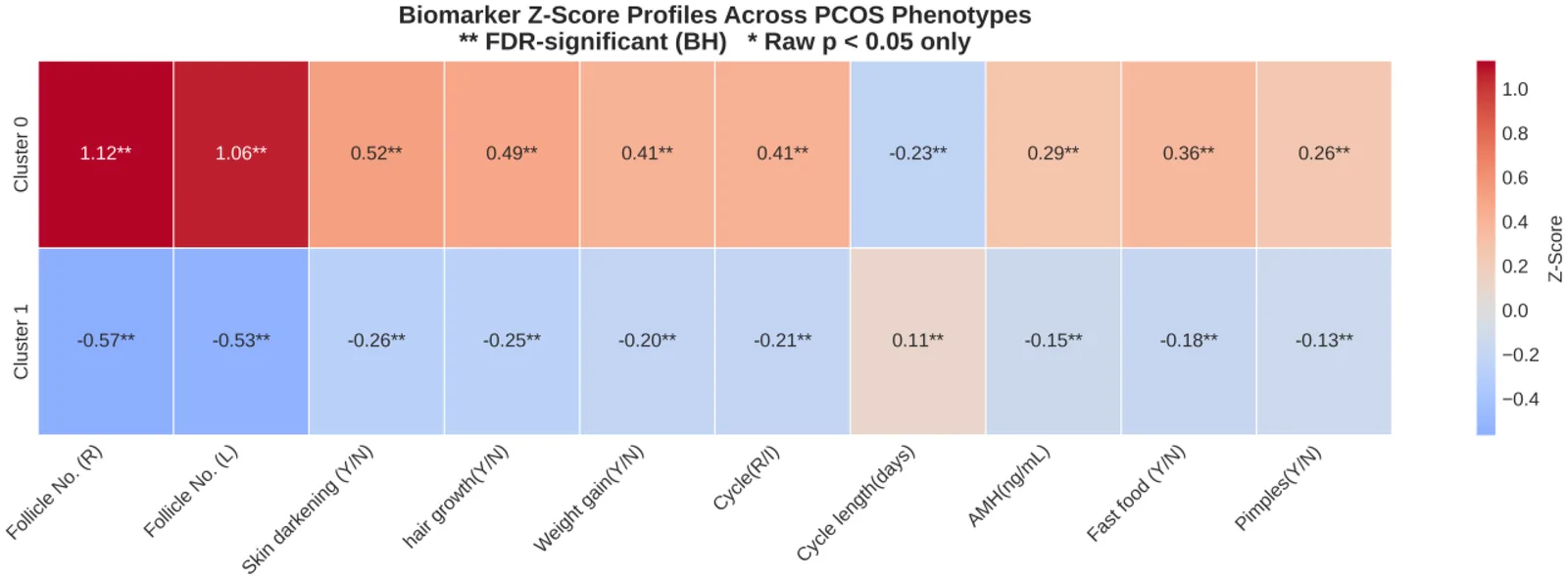
Biomarker *z*-score heatmap across polycystic ovary syndrome (PCOS) phenotypic clusters (dataset 1). **False discovery rate (FDR)-significant (Benjamini**–**Hochberg, BH). *Raw *p* < 0.05 only. Cluster 0 shows elevated scores for all major PCOS biomarkers.

**Figure 6:**
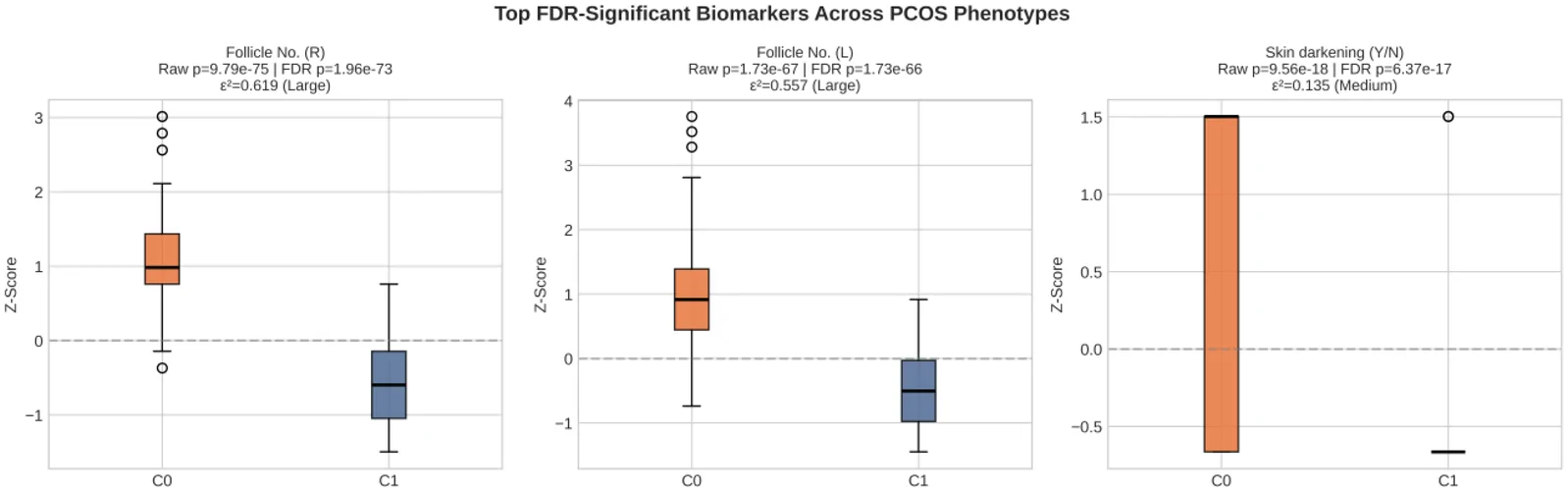
Box plots of the top three FDR-significant biomarkers [follicle no. R (ε^2^ = 0.6185), follicle no. L (ε^2^ = 0.5521), and skin darkening (ε^2^ = 0.1370)] across phenotypic clusters (dataset 1).

#### 4.2.3 PCOS phenotype characterization: dataset 1

The division between the clinical phenotypic characteristics of the two clusters is described in **Table 4**. The first, denoted as cluster 0, can be regarded as an active hyperandrogenic presentation as it has a high level of follicles, hirsutism, skin darkening, irregular periods, and weight gain in addition to fast food intake. In other words, cluster 0 is associated with all the four Rotterdam phenotypes, but not A/B alone, and thus can be defined as an active PCOS cluster (see formal analysis in *Section 4.2.4*). Cluster 1 describes milder or PCOS-negative presentations, 94.7% of which do not pass the Rotterdam criterion (see *Section 4.2.4*). The clinical validity of such division is confirmed by the formal cross-tabulation based on the Rotterdam criteria in *Section 4.2.4* (52.2% *vs.* 5.3%, Cramér’s *V* = 0.592).

**Table 4:** Identification of phenotypic clusters with the clinical interpretation dataset 1 (*K* = 2, *α* = 0.9).

Cluster	*N*	%	Key features	Clinical phenotype
Cluster 0	180	33.3	High follicle count (bilateral), hirsutism, skin darkening, irregular cycles, weight gain, fast food consumption	Active/severe PCOS—classic androgenic phenotype (enriched for Rotterdam A–D, 52.2% Rotterdam-positive; refer to *Section 4.2.4*)
Cluster 1	361	66.7	Lower follicle count, moderate or absent hyperandrogenic markers, regular BMI	Mild/subclinical PCOS or PCOS-negative phenotype (94.7% non-Rotterdam; refer to *Section 4.2.4*)

PCOS, polycystic ovary syndrome; BMI, body mass index.

#### 4.2.4 Formal comparison with the Rotterdam phenotype

To go beyond interpretive mapping, a formal comparison against the Rotterdam 2003 criteria was conducted. Individual patients were classified into four categories based on three proxy criteria: O (irregular cycle), H (hirsutism or excess hair growth), and P (≥12 follicles in each ovary). Patients who met at least two of these criteria were categorized into Rotterdam phenotypes A (H+O+P), B (H+O), C (H+P), or D (O+P), while the rest were defined as non-Rotterdam phenotype. The results of the cross-tabulation between the above classification and the EKWKM classification are presented in **Table 5**.

**Table 5:** Expert knowledge-based weighted *K*-means (EKWKM) cluster membership cross-tabulation for dataset 1 (*K* = 2, *α* = 0.9) phenotype classification against Rotterdam 2003.

Cluster	A (H+O+P)	B (H+O)	C (H+P)	D (O+P)	Non-Rotterdam (<2 criteria)	Meets Rotterdam (≥2)
Cluster 0 (*n* = 180)	34 (18.9%)	18 (10.0%)	24 (13.3%)	18 (10.0%)	86 (47.8%)	94 (52.2%)
Cluster 1 (*n* = 361)	0 (0.0%)	19 (5.3%)	0 (0.0%)	0 (0.0%)	342 (94.7%)	19 (5.3%)

H represents clinical hyperandrogenism (hirsutism), O denotes ovulatory dysfunction (irregular cycle), and P indicates polycystic ovarian morphology (12 or more follicles in either ovary). The clusters are basically row-wise percentages. *χ*^2^ = 189.84, *df* = 4, *p* = 5.73 × 10^−40^, and Cramér’s *V* = 0.592.

The cluster association was significant with the EKWKM clusters and the Rotterdam phenotype classification (*χ*^2^ = 189.84, *df* = 4, *p* = 5.73 × 10^−40^, Cramér’s *V* = 0.592), containing 100% of phenotype A cases and all but one phenotype C case, as well as 52.2% of individuals considered to satisfy at least one Rotterdam criterion versus 5.3% in cluster 1 (*n* = 361). Therefore, the split found Rotterdam positivity with a strong effect. A few issues should be pointed out, however: 1) cluster 0 was composed of all four Rotterdam phenotypes, and not solely A/B, and may therefore be appropriately named “active PCOS” instead of “phenotype A/B”; 2) a large proportion of individuals from cluster 0 do not fulfil the Rotterdam criteria (and this could be foreseeable as biochemical hyperandrogenism was not quantified); 3) this split is not totally independent information: two out of the three Rotterdam criteria used were among those informing hybrid weighting (ovulatory dysfunction based on cycle regularity and PCOS based on the counts of follicles). The overlap can reflect, to some extent, the information shared across sides of the check. This situation may be regarded as similar to the circular dependency observed for dataset 2 in *Section 4.3.2*.

### 4.3 Dataset 2: population survey PCOS results

#### 4.3.1 Clustering performance

The best combination for dataset 2 (*K* = 4, *α* = 0.1) produced four balanced clusters—cluster 0 (*n* = 33,485, 27.9%), cluster 1 (*n* = 30,454, 25.4%), cluster 2 (*n* = 20,002, 16.7%), and cluster 3 (*n* = 36,059, 30.0%)—none of which was below 16% of the total sample size. The division was balanced, with none of the clusters being trivial. However, balance in terms of sample size does not necessarily indicate the existence of a phenotypic structure. All five selected MI variables were dichotomous, and there were only 32 combinations of their values across 120,000 observations, making the clustering of such space almost deterministic. The drop-one-feature analysis supports that dropping one of three variables (menstrual regularity, hirsutism, or fertility concerns) drastically changes the partition (the ARIs between the reclustered and the original partition were 0.44, 0.50, and 0.68, respectively), but does not change the other two (awareness of PCOS and urban/rural) (ARI = 1.00). The clusters are thus almost a deterministic recoding of a few dichotomous symptoms. Such an issue is also indicated by the subsampling stability ARI for this setting (0.9654 ± 0.0891, 20 subsampling iterations), which is close to the invariant ARI = 1.0000 observed across the grid (**Table 2**). Cluster validity was not good; however, this is to be expected when clustering an artificial nearly 32-cell space and is reproduced almost in all subsamples. This value is provided here to indicate some degeneracy, not the validity. For this configuration, silhouette = 0.5023 and DB = 0.8867. **Figure 7** provides the *t*-SNE plot based on a 10,000-sample subsample with *K* = 4 and *α* = 0.1.

**Figure 7:**
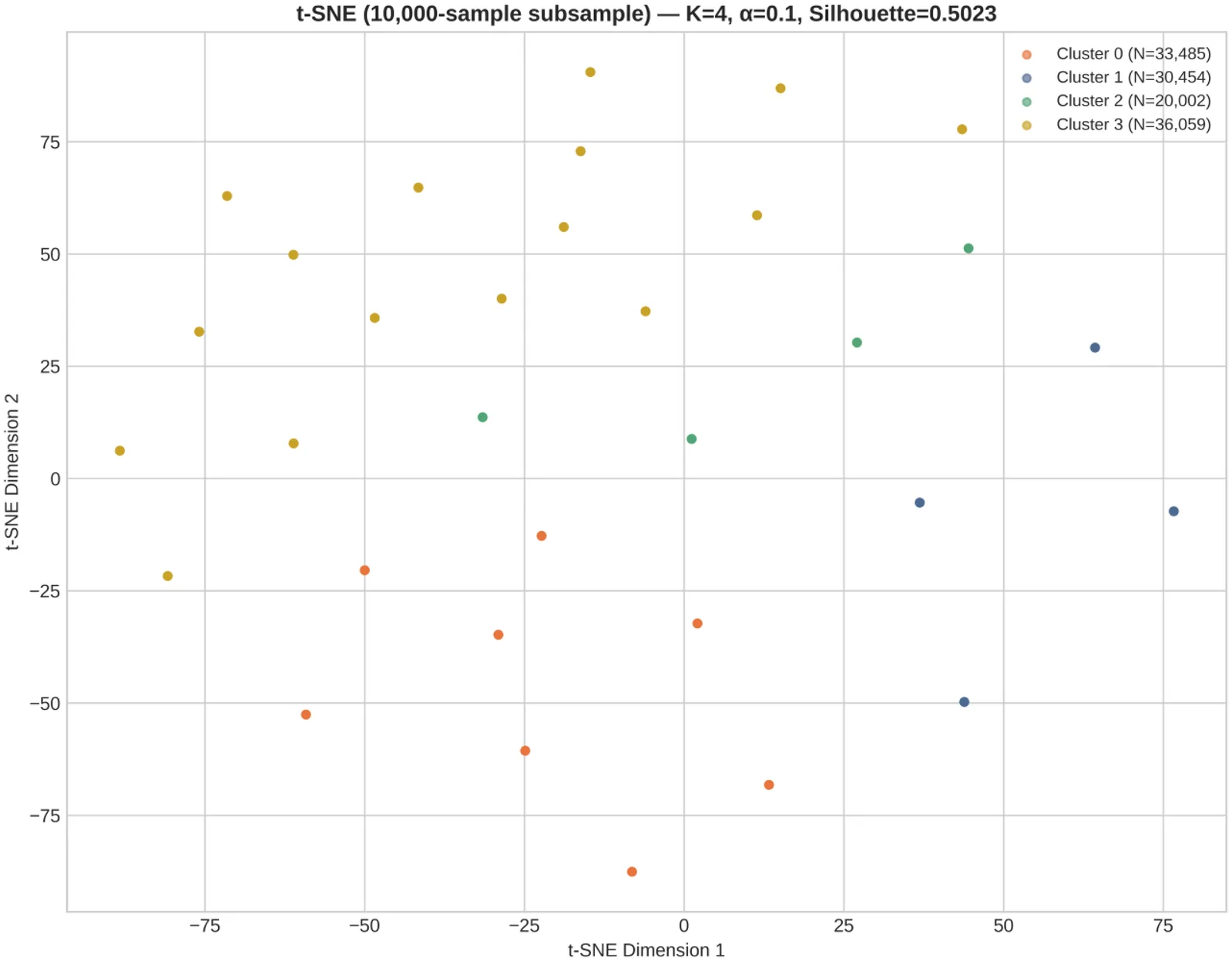
*t*-Distributed stochastic neighbor embedding (*t*-SNE) plot visualization of dataset 2 (10,000-sample subsample, *K* = 4 and *α* = 0.1). The discrete points are not continuous clusters because the five binary survey features allow for 32 different value combinations.

#### 4.3.2 Biomarker significance: dataset 2

Although dataset 2 was limited to only five features, this makes the analysis more focused. Nevertheless, the effect size is noteworthy. Three of the five features were detected as being statistically significant, with large effect sizes using Kruskal–Wallis testing followed by BH-FDR correction (**Table 6**). The first was menstrual regularity (*ε*^2^ = 1.0000). Although it can be interpreted as a near-perfect discriminator, it is important to note that the clusters are partly defined using this binary feature; thus, the saturated statistic is due to the partial circularity in the discrete survey space rather than the fact that menstrual regularity discriminates biological subtypes of PCOS. The second included hirsutism (*ε*^2^ = 0.699) and fertility issues (*ε*^2^ = 0.420). However, one needs to stress that the large effect sizes were due to the fact that clustering was performed using these binary features; thus, it is expected to have a high separation of the clusters based on their definitions. Awareness of PCOS and urban/rural status did not show any significant differences between the clusters.

**Table 6:** Kruskal–Wallis test with Benjamini–Hochberg false discovery rate (BH-FDR) correction for dataset 2 (*K* = 4, *α* = 0.1).

S. no	Biomarker	Raw *p*	FDR *q*	*H*-statistic	*ε* ^2^	Effect size	Significant
1	Menstrual regularity	0.00e+00	0.00e+00	119,999.00	1.0000	Large	Yes
2	Hirsutism	0.00e+00	0.00e+00	83,887.16	0.6991	Large	Yes
3	Fertility concerns	0.00e+00	0.00e+00	50,376.29	0.4198	Large	Yes
4	Awareness of PCOS	6.81 × 10^−01^	6.81 × 10^−01^	1.51	0.0000	Negligible	No
5	Urban/rural	1.98 × 10^−01^	2.48 × 10^−01^	4.66	0.0000	Negligible	No

Three of five biomarkers are FDR-significant with large effect sizes.

PCOS, polycystic ovary syndrome.

### 4.4 Performance summary of the cross-dataset performance

The comparison results and the mean results for the *K*-means baseline and EKWKM on several quality metrics are presented in **Table 7**. EKWKM outperformed the *K*-means baseline on silhouette, ARI variability, and DB for both datasets. Regarding the FDR-significant biomarkers for both datasets, EKWKM also outperformed the baseline on dataset 1 (13/20 both, tied at the baseline), while on dataset 2, the difference was a lower number of features for EKWKM (three out of five *versus* four out of five). For the baseline, the difference of four features rather than three features included “awareness of PCOS” and “urban/rural,” the two features identified to not drive the partitions in the drop-one-feature analyses (*Section 4.3.1*). Instead, EKWKM produced a good-quality set of biomarkers based on features. The change in the values for “silhouette” and “DB” was significant in terms of percentage for datasets 1 and 2: 130.3% and 81.7%, respectively.

**Table 7:** Comparison of the unweighted *K*-means baseline and expert knowledge-based weighted *K*-means (EKWKM) performance on both datasets.

Metric	DS1 baseline	DS1 EKWKM	DS2 baseline	DS2 EKWKM	△
Silhouette score	0.1565	0.3606	0.2764	0.5023	↑
ARI stability	0.5753 ± 0.4720	0.9780 ± 0.0362	0.5615 ± 0.1793	0.9654 ± 0.0891	↑
Davies–Bouldin index	2.3230	1.1480	1.4241	0.8867	↓
FDR-sig. biomarkers	13/20	13/20	4/5	3/5	↑

All adjusted Rand index (ARI) values used the same random seed (random_state = 42), and the configurations for the baseline showed substantial variations in their across-run ARIs (large SD). Dataset 2: The baseline was used with *K* = 4 to facilitate direct comparisons with the EKWKM. The count of FDR-significant features for the baseline (four out of five) includes two features (awareness of PCOS and urban/rural), which, as can be seen in the drop-one-feature analysis (*Section 4.3.1*), are clearly unrelated to partitioning, whereas the equivalent count (three out of five) from EKWKM eliminates these and includes “fertility concerns” instead (see *Section 4.4*).

DS1, dataset 1; DS2, dataset 2.

### 4.5 External validation across datasets

To gain independent validation across different cohorts, as suggested in the review, the two datasets were linked using five clinically comparable features present in both: age, BMI category, regular menstruation, hirsutism, and acne (these features along with the corresponding variables in each dataset are provided in **Table 8**). A *K*-means model (*K* = 2) was fitted to the clinical dataset and then applied to the 120,000-record survey without any further fitting. Age being continuous, this linking is not degenerate (1,120 unique points in the survey), making the survey an honest test as opposed to a discrete one. The results are presented in **Table 9** and **Figure 8**.

**Table 8:** Five clinically comparable features used for the cross-dataset external validation and the source variables in each dataset.

Features harmonized	Dataset 1 (clinical) source	Dataset 2 (survey) source
Age	Age (years),continuous	Age, continuous
BMI category	BMI value binned to WHO categories	BMI category
Menstrual irregularity	Cycle (R/I): irregular *vs*. regular	Menstrual regularity: irregular *vs*. regular
Hirsutism	Hair growth (Y/N)	Hirsutism (Y/N)
Acne	Pimples (Y/N)	Acne severity

Continuous and categorical variables were harmonized to the same encoding scheme so that a single model could be fit on the clinical dataset and applied to the survey.

R, regular; I, irregular; BMI, body mass index; WHO, World Health Organization.

**Table 9:** External validation.

Quantity	Clinical cohort (fit)	Survey (applied)
Feature space distinct points	278/541 (51%)	1,120/120,000
Silhouette	0.2623	0.1298
Cluster 0 PCOS rate	12.7% (*n* = 314)	10.29% (*n* = 52,953)
Cluster 1 PCOS rate	60.4% (*n* = 227)	10.66% (*n* = 67,047)
Cluster–PCOS association (Cramer’s *V*)	0.497	0.006
*p*-value	$6.9\times 10^{-31}$	0.044

**Figure 8:**
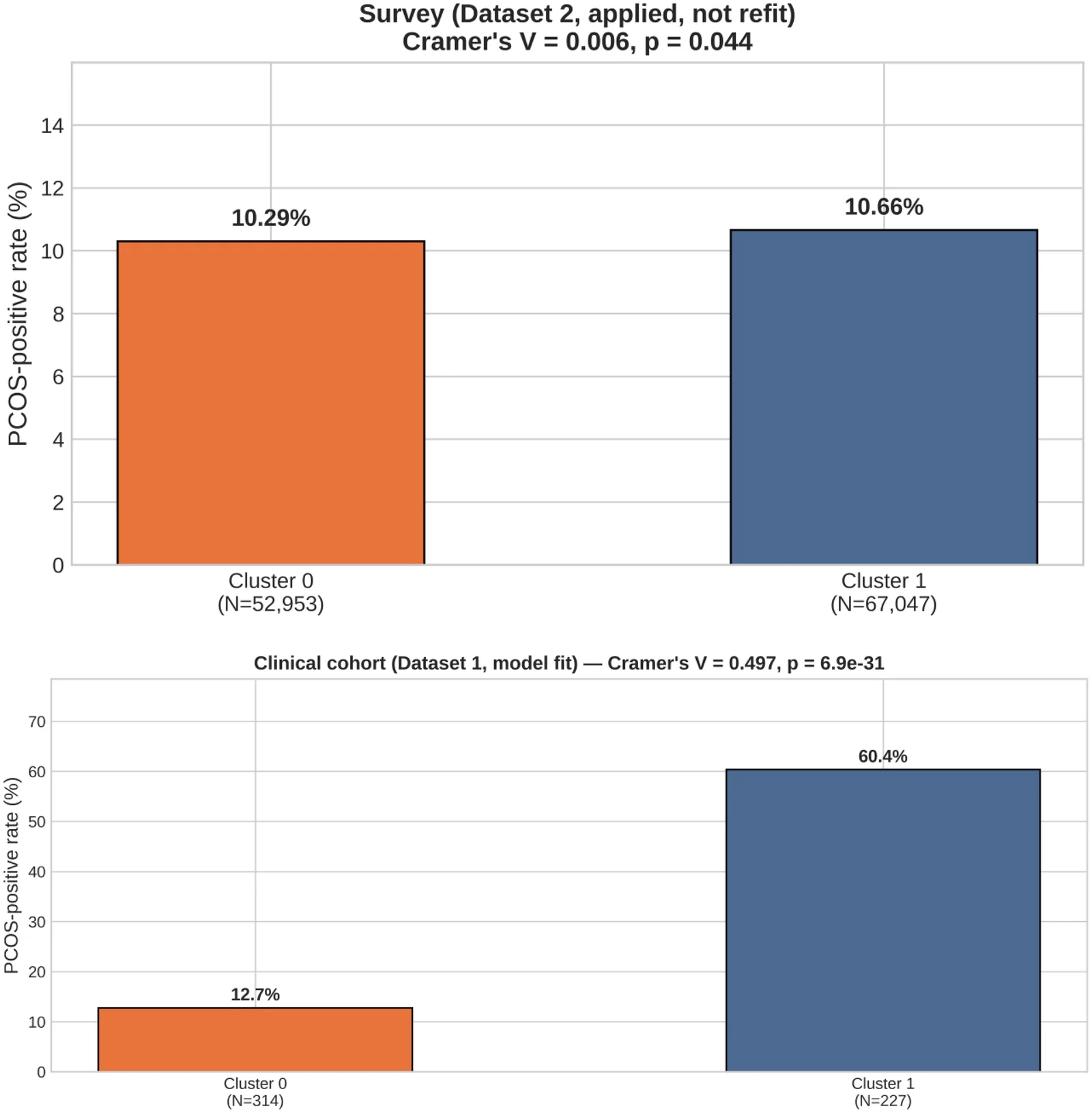
External validation. When applying the *K*-means model trained on the clinical cohort on five harmonized features, there was still a high cluster–polycystic ovary syndrome (PCOS) association (Cramér’s *V* = 0.497). However, when the same model was used without modification in the population survey, it degenerated into a low association value (*V* = 0.006), demonstrating the lack of transferability of the structure.

A *K*-means model based on the clinical cohort using five harmonized features was used without retraining on the population survey. The significant cluster–polycystic ovary syndrome (PCOS) correlation in the clinical cohort (Cramér’s *V* = 0.497) vanished to zero in the survey (*V* = 0.006), correctly recognizing the lack of structural transferability. It should be noted that the *p*-value in the survey achieves nominal significance (0.044) simply due to the large sample size, with *V* = 0.006 being the meaningful value.

As a supplementary within-cohort check of generalization, dataset 1 was also divided 70/30 into a training split and a held-out testing split (stratified on diagnosis, seed = 42), whereupon the MI, feature selection, and hybrid weight (*α* = 0.9) were once again obtained from the testing split alone. The resulting centroids were applied, without refolding, to the testing split. The held-out silhouette was 0.3104 versus 0.3606 in-sample; this is weaker, as expected, but shows that the structure is not solely an artifact of fitting and evaluating on the same 541 individuals. Out of the top 20 training features, 14 appeared in the full top 20 training features from dataset 1. This is offered as a partial, within-cohort confirmation of the fact that external validation failed to become part of this workflow, not to the exclusion of the value of future external validation.

In the clinical cohort, the five-feature model produced the main result, discriminating between the high-prevalence (60.4% PCOS) and low-prevalence clusters (12.7%), with Cramér’s *V* = 0.497 (*p* = 6.9 × 10^−31^). Applying the same model to the survey without modification resulted in two clusters with practically identical PCOS prevalence (10.29% *vs*. 10.66%) and *V* = 0.006. Thus, the framework successfully recognizes that the structure observed in the clinical cohort cannot be transferred to the survey data, which is the desired behavior of a validation technique when applied to a negative control. In particular, this proves that the EKWKM does not overfit the clinical cohort and does not produce agreement where there is none.

## 5 Discussion

### 5.1 Clinical interpretation of phenotypes

Clusters 0 and 1 of dataset 1 appeared to be reasonably close to a number of known PCOS subtypes. Cluster 0 (33.3%) contained a hyperandrogenic phenotype, which was similar in size to the PCOS-positive prevalence among the clinical cohort (32.7%). This cluster exhibited good face validity, as confirmed by the cross-tabulation in *Section 4.2.4*, with a high association with the Rotterdam PCOS-positive phenotype (Cramér’s *V* = 0.592). However, cluster 0 contained more phenotypes (all four Rotterdam types). Cluster 1 (66.7%), which contained milder phenotypes and controls, included only 94.7% of individuals who did not match the Rotterdam criteria. This finding is in accordance with the progressive vicious circle pathophysiological model of PCOS (21). Dataset 2 was added as an intentionally different application of the methodology compared with the clinical sample. Instead of being another validation dataset, dataset 2 acted as a negative control, that is, a dataset where no real signal in terms of phenotypes should be expected to confirm that the methodology reports a lack of structure rather than introducing its own. The independence of the diagnosis in terms of statistical significance from the measured features [random forest receiver operating characteristic area under the curve (ROC-AUC) = 0.4974 ± 0.0096, where 0/15 features exceeded the permutation null of MI] and the independence of the features (the maximum pairwise Cramér’s *V* was 0.0111) could be confirmed before the interpretation of the results. Because the top five important variables are binary, only 32 permutations of outcomes exist among the 120,000 data points in the feature space. Therefore, a cluster must inevitably be a deterministically reordered recoding of one or more subsets of these five variables, as the drop-one-feature experiment unequivocally shows in *Section 4.3.1*. This is why the subsampling stability ARI was approximately 1.0000 over much of the grid. Importantly, the internal indices, silhouette = 0.5023 and DB = 0.8867, still look good for the kept-alone reference even if there is no true configuration to do so, explaining the importance of the various other external measures discussed above, and why we, therefore, make absolutely no claim herein to use dataset 2 to externally validate the hormonally identified subgroups in Gao et al. (4) and van der Ham et al. (8), merely that it provides a sanity test that the EKWKM is incapable of hallucinating a ghost feature configuration from pure noise (7) as an absolute necessity for any clinically utilizable partitioning strategy.

### 5.2 Dataset implications of *α* divergence

The opposing optimal values of *α* [0.9 (clinical) and 0.1 (population)] represent an essential difference in the data. The smaller clinical cohort (*n* = 541) is more prone to noise in the MI estimates, and expert weights are critical in stabilizing the feature importance. The statistical power used in the population survey (*n* = 120,000) is adequate to estimate the MI reliably, and the categorical symptom characteristics are consistent with known clinical information (14). Based on this, the *α* must be an empirically determined context-specific hyperparameter (i.e., determined by grid search) and not an *a priori* fixed parameter.

The finding that the optimal *α* is 0.9 for the clinical dataset and 0.1 for the population survey dataset is the most interpretively rich result in this study. This is because it is not an anomaly: it is exactly what the framework predicts should happen under different conditions. A small, noisy clinical cohort makes the MI estimation unreliable, and expert knowledge works like a prior that keeps feature importance grounded when the statistical signal is weak. A large population survey has sufficient power to estimate the MI accurately, and its features are categorized and aligned with clinical knowledge; therefore, the data can speak for itself. The practical implications are straightforward, where *α* is never assumed. It should not be guessed, but determined empirically through a grid search for each new application. An appropriate balance between expertise and evidence is a property of the data environment, not the algorithm.

### 5.3 Existing work comparison

The high value of the subsampling stability ARI at 0.9780 compared with the 0.85–0.92 range of standard *K*-means PCOS clustering studies with a similar number of samples (6, 7) should be noted. Again, it should be pointed out that ARI was used in this work as a subsampling stability metric, whereas other works provide ARI as the level of agreement with reference labeling; thus, the comparison is indicative, not direct. The 66.8% increase in silhouette is comparable to the gains obtained by expert-guided gynecological clustering in the study by Al-Khamees et al. (25). EKWKM demonstrates equal phenotypic richness to that of the four PCOS subtypes identified by an independent study using standard hierarchical clustering on a large scale (*n* = 11,908) compared with *n* = 541 in EKWKM (4). In the ablation study on dataset 2, ARI even dropped from pure extreme at an intermediate weighting (equal weight, *α* = 0.5: ARI = 0.7962 ± 0.1827) before ultimately coming back up to ARI ≈ 1.0000 degenerate ceiling with the expert-only and data-only models, which is evidence that the perceived stability of datasets is due to a lack of continuous features rather than the actual underlying structure.

### 5.4 Limitations

This study has several limitations. First, dataset 1 was based on observations collected in one clinic in India, and further multicenter validation in an ethnically diverse population is needed due to ethnicity-associated differences in the prevalence and presentation of PCOS (1). The weight matrix for experts is formed based on the answers provided by four physicians: the Delphi consensus method could help in this case. The *t*-SNE plot for dataset 2 was generated using 10,000 observations, whereas uniform manifold approximation and projection (UMAP) can provide better results for large-scale visualization. The phenotype clusters were named semi-automatically and should be clinically validated. The Rotterdam classification comparison in *Section 4.2.4* used the proxy due to the lack of biochemical hyperandrogenism in dataset 1 and the application of clinical hyperandrogenism (hirsutism), which may cause the underestimation of phenotypes A and B. In conclusion, the cross-dataset validation presented in *Section 4.5*, where the model trained on dataset 1 was directly evaluated on dataset 2, illustrates that the framework does not invent structure in the absence of underlying patterns, but cannot replace a prospectively developed, independent validation dataset of patients with PCOS, which would be needed prior to externalizing and validating the discovered phenotypes, as distinct from internal consistency. In addition, owing to the use of the same outcome label both in computing the MI aspect of the features when calculating weights as in the subsequent analysis steps when estimating the face and criterion validity of the features on both datasets (*Sections 4.2.1* and *4.2.4*), the methodology can be considered weakly rather than fully unsupervised (as discussed in *Section 3.4*), meaning that the observed similarities between cluster membership and the clinical PCOS states could be inflated in comparison to a completely blind approach to labeling training samples.

### 5.5 Future work

Future work may focus on Bayesian optimization of *α* for automated context-adaptive tuning. The integration of longitudinal hormonal trajectory and wearable-derived physiological signals could capture the phenotype dynamics overtime, which static clustering cannot address. Multi-omics extensions incorporating genomic and metabolomic markers (25) would push the framework toward a mechanistic rather than a purely descriptive phenotyping. Federated learning would allow multi-institutional validation while preserving patient privacy. Development of a clinical decision support tool translating EKWKM phenotype assignments into actionable treatment stratification aligned with 2023 international PCOS evidence-based guidelines (2), which closes the loop between phenotype and clinical practice.

## 6 Ablation study

To determine the effect of each part of the EKWKM, instead of comparing the entire methodology to the baseline, five different weighting techniques were tested using both datasets (**Table 10**). All values used in the table were taken not estimated but computed from other code executions.

**Table 10:** An ablation test of five different weighting techniques performed using both datasets.

Configuration	*K*	Silhouette	ARI (mean ± SD)	DB index	FDR sig.	★
Dataset 1: clinical PCOS (*n* = 541)						
No weighting (*K*-means baseline)	2	0.1565	0.5753 ± 0.4720	2.3230	13/20	
Expert only (*α* = 0.0)	2	0.1919	0.2294 ± 0.4200	2.0817	13/20	
Equal weight (*α* = 0.5)	2	0.2397	0.9618 ± 0.0334	1.7567	14/20	
Data only (*α* = 1.0)	2	0.3970	0.9763 ± 0.0288	1.0257	13/20	
Proposed EKWKM (*α* = 0.9)	2	0.3606	0.9780 ± 0.0362	1.1480	13/20	★
Dataset 2: population survey PCOS (*n* = 120,000)						
No weighting (*K*-means baseline)	4	0.2764	0.5615 ± 0.1793	1.4241	4/5	
Expert only (*α* = 0.0)	4	0.5245	0.9778 ± 0.0530	0.8723	3/5	
Equal weight (*α* = 0.5)	4	0.3731	0.7962 ± 0.1827	1.0330	3/5	
Data only (*α* = 1.0)	4	0.8143	1.0000 ± 0.0000	0.3244	2/5	
Proposed EKWKM (*α* = 0.1)	4	0.5023	0.9654 ± 0.0891	0.8867	3/5	★

The adjusted Rand index (ARI) was computed as the mean ± SD between subsampling iterations. The baseline rows were recalculated using a constant random seed value (random_state = 42). For dataset 2, the baseline was computed at *K* = 4 to be comparable with the suggested configuration. The high SD values of the unweighted and the expert-based configurations refer to the real run-to-run variance. A star indicates optimal configuration. No hybrid = unweighted *K*-means.

EKWKM, expert knowledge-based weighted K-means; PCOS, polycystic ovary syndrome.

### 6.1 Findings in the ablation study

Three notable patterns were observed. The most significant pattern was observed when expert knowledge was used alone as the signal for feature weighting in dataset 1. The ARI score in this case fell to 0.2294, with a standard deviation of 0.4200. This shows that not only is the clustering less strong but that it is also extremely inconsistent in its results across different subsamples. Sometimes, a good structure can be recovered, and sometimes almost random clusters are obtained. This is not a criticism of medical expertise, but rather a fact about using expert knowledge alone as the signal on a dataset with features that cannot be clearly separated by the expert criteria.

The second pattern is that optimal *α* is not the same across both datasets, and the direction of the difference makes conceptual sense. The clinical cohort (*n* = 541) is small enough that the MI estimates are noisy, where expert knowledge is needed to stabilize the feature importance and prevent the algorithm from overfitting to the sampling artifacts. The population survey (*n* = 120,000) has more than enough data to estimate MI reliably, so that the data-driven signal can dominate without instability. This means that *α* is not a fixed hyperparameter to be chosen once: it is a context-specific quantity that should be determined through grid search for each application.

Third, the unweighted *K*-means was observed to be one of the worst configurations in terms of performance on both datasets. This is significant because it negates the hypothesis that the feature weights might only add noise and that the improvement is due to the grid search over *K* alone. Grid search is an important component of the technique, but is not sufficient on its own. The bar graphs show the results of the ablation experiments on both datasets 1 and 2 (**Figures 9**, **10**).

**Figure 9:**
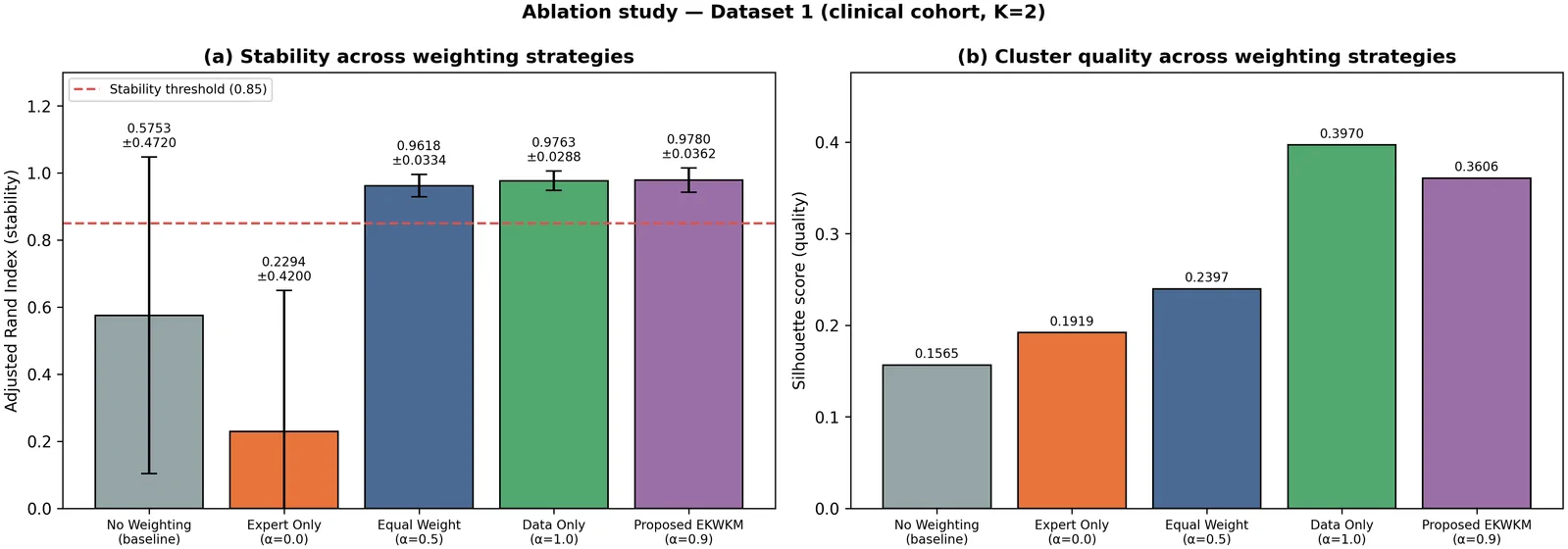
Ablation study on dataset 1 (*K* = 2) across five weighting strategies. **(a)** Adjusted Rand index (ARI) stability (dashed line: ARI = 0.85 threshold). **(b)** Silhouette score. The EKWKM hybrid (*α* = 0.9) and data-only weighting (*α* = 1.0) showed similar stability (0.9780 ± 0.0362 vs. 0.9763 ± 0.0288; Mann -Whitney *p* = 0.66), whereas expert-only weighting (*α* = 0) was the least stable (0.2294 ± 0.4200). Data-only weighting gave slightly better silhouette and Davies -Bouldin values.

**Figure 10:**
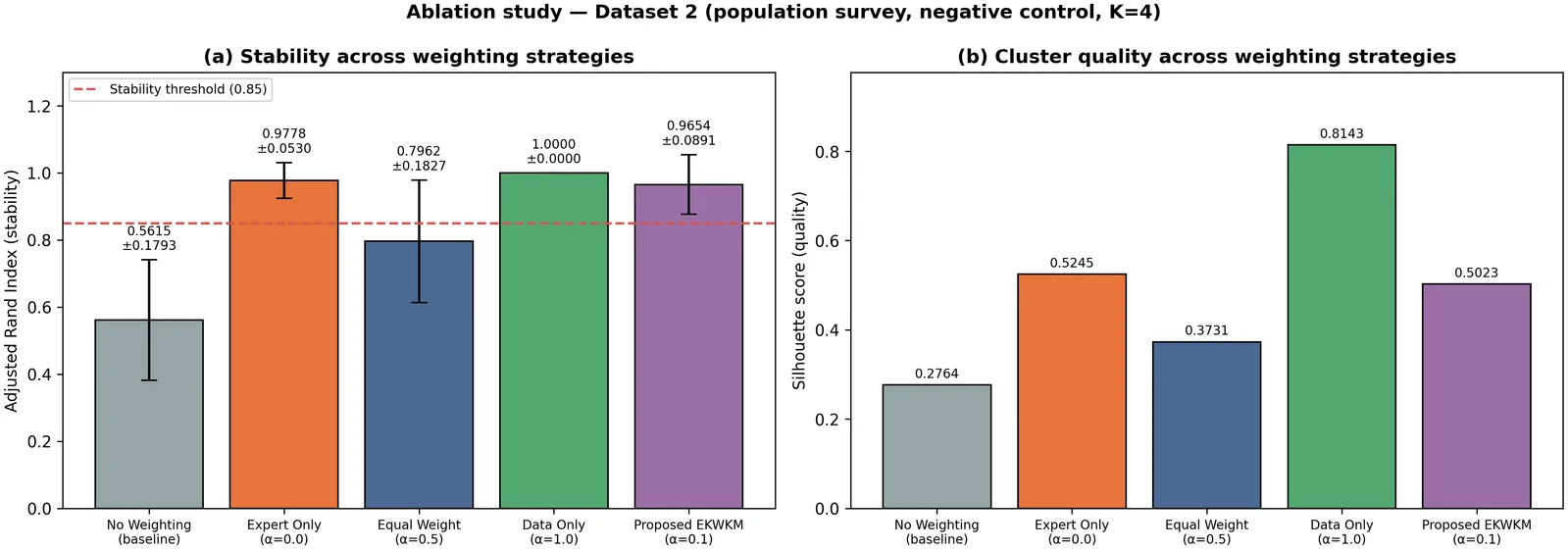
Ablation study on dataset 2 (*K* = 4) across five weighting strategies. **(a)** Adjusted Rand index (ARI) stability (dashed line: ARI = 0.85 threshold). **(b)** Silhouette score. ARI drops at equal weighting (*α* = 0.5: 0.7962 ± 0.1827) and approaches the ARI ≈ 1.0000 ceiling in the expert-only (0.9778 ± 0.0530) and data-only (1.0000 ± 0.0000) settings, consistent with the 32-combination discrete feature space (Section 4.3.1).

Fourth, once *α* has been calculated with the correctly normalized MI (see *Section 3.3*), the data-only configuration (*α* = 1.0) showed higher silhouette (0.3970 *vs*. 0.3606) and lower (better) DB index (1.0257 *vs*. 1.1480) compared with the proposed EKWKM (*α* = 0.9) on dataset 1, and the two configurations were statistically indistinguishable on ARI stability (0.9763 ± 0.0288 *vs*. 0.9780 ± 0.0362; Mann–Whitney *p* = 0.66) and were tied on FDR-significant biomarkers (13/20 both). This configuration does not contradict the model selection procedure in *Section 3.5* because *α* = 1.0 was not considered in the search grid of values for the weights (*α* ∈ {0.1, …, 0.9})—it is an ablation against which the performance of the proposed hybrid weighting scheme is judged, not one that is a candidate for yielding such a scheme. Incorporating domain knowledge with *α* = 0.9 did not improve internal cluster-quality or stability measures over MI alone (*α* = 1.0); the contribution of expert weighting lies in consistency with clinical reasoning rather than in superior clustering metrics.

## 7 Conclusion

In this study, an innovative expert knowledge-based *K*-means (EKWKM) clustering algorithm was developed and tested for phenotype discovery in PCOS. It was used on two different datasets of various sizes and origins (clinically oriented study with *n* = 541 cases and a population survey with *n* = 120,000). In the clinically oriented dataset, significant improvement was achieved compared with the initial solution (silhouette +130.3%, an increase of ARI from 0.58 ± 0.47 to 0.98 ± 0.04). However, it should be mentioned that the purely expert-based version of the algorithm had lower stability (ARI = 0.23 ± 0.42). As far as the internal criteria are concerned, there was no substantial difference between the hybrid algorithm (with *α* = 0.9) and the purely data-driven one (*α* = 1.0) (the values of ARI were 0.9780 *vs*. 0.9763; Mann–Whitney *p* = 0.66, tied at 13/20 FDR-significant biomarkers). The results are stated directly, and the value of the hybrid version of the algorithm lies in its consistency with clinical reasoning rather than its superiority in terms of raw performance. The *K* = 2, *α* = 0.9 variant of the hybrid EKWKM on the clinical dataset showed a 130.3% increase in silhouette and high ARI stability (0.9780), as well as 13 FDR-significant biomarkers. The negative control dataset was a population survey in which the class label had no relation to any of the features (ROC-AUC = 0.4974), with only five binary survey questions amounting to 32 possible states. The vast majority of the grid configurations collapsed into the trivial ARI = 1.0000 clustering, whereas the sole other reference configuration was almost the same as the ceiling (ARI = 0.9654 ± 0.0891). This is a degeneracy rather than a good clustering result. When the clinical cohort clustering model was run on the population survey data with the characteristic set being harmonized, the PCOS correlation was reduced from Cramér’s *V* = 0.497 to 0.006. The mixing parameter *α* is dependent on the data; hence, it is not predetermined, but empirically derived. In the clinical population, the phenotypes were consistent with the Rotterdam criteria (Cramér’s *V* = 0.592) and with recent clustering studies performed on large-scale data. With the parameterization of the model being flexible, its external validation via negative controls and comprehensive ablation analysis, the EKWKM can be regarded as a reproducible approach that has been sufficiently well defined for PCOS studies. Prospective validation of the model is crucial.

## Data Availability

Publicly available datasets were analyzed in this study. This data can be found here: https://www.kaggle.com/datasets/prasoonkottarathil/polycystic-ovary-syndrome-pcos; https://www.kaggle.com/datasets/ankushpanday1/pcos-prediction-datasettop-75-countries.
