# Supplementary material for: Expert knowledge-based weighted K-means clustering for polycystic ovary syndrome phenotype identification
Source: Front Endocrinol (Lausanne). 2026 Sep 30;17:1871986. doi: 10.3389/fendo.2026.1871986 (PMC13662547; doi:10.3389/fendo.2026.1871986)
Supplement: Supplementary file 1 [file DataSheet1.pdf]

Request for Expert Opinion on PCOS Dataset Feature Weighting

From

D. Priyanka [23PHD0266],
Research Scholar,
SCORE, VIT, Vellore.
priyanka.d@vit.ac.in
9597036629

To

The Medical Officer,
Sri Narayani Hospital and Research Centre,
Vellore.

Subject: Seeking Expert Opinion on PCOS Risk Factors & Feature Weighting for Research.

Dear Dr. Devamani Pandiyan ,MBBS, MS(OG), M. Med SCI (UK),

I hope this letter finds you well. I am D. Priyanka, a research Scholar/PhD Student at VIT Vellore, working under the guidance of Dr.Sumangali.K, Associate Professor ,SCORE . I am currently conducting a research study on **Polycystic Ovary Syndrome (PCOS) prediction and diagnosis through machine learning and Deep Learning techniques.**

As part of this study, I aim to assign **relative importance (weights) to key clinical and biochemical features** related to PCOS, based on expert medical knowledge. Your insights as a specialist in [Endocrinology/Gynaecology/Related Field] would be invaluable in ensuring the accuracy and reliability of the feature selection process.

I have two datasets related to PCOS and prepared a table listing **43 features and 17 features**, including **BMI, hormonal levels, ultrasound findings, and lifestyle factors.** I kindly request your expert opinion on assigning **weights (1-10)** to each feature based on its significance in diagnosing or predicting PCOS.

Your feedback will play a crucial role in refining the feature selection process and improving the **clinical applicability of AI-based PCOS prediction models.** Please find the table attached for your review. I sincerely appreciate your time and expertise in supporting this research.

Looking forward to your valuable insights.

Thanking you,

Sincerely,

D. Priyanka [23PHD0266],
Research Scholar,
SCORE, VIT, Vellore.

Under the Guidance of

Dr. Sumangali .K,
Associate Professor,
SCORE, VIT, Vellore.

Dataset 1

Sl. No.	Feature	1	2	3	4	5	6	7	8	9	10
1	Patient File No.	☑	☐	☐	☐	☐	☐	☐	☐	☐	☐
2	PCOS (Y/N)	☐	☐	☐	☑	☐	☐	☐	☐	☐	☐
3	Age (yrs)	☐	☐	☐	☐	☐	☐	☐	☐	☐	☑
4	Weight (Kg)	☐	☐	☐	☐	☐	☐	☐	☐	☐	☑
5	Height (Cm)	☐	☐	☐	☐	☐	☐	☐	☐	☐	☑
6	BMI (Body Mass Index)	☐	☐	☐	☐	☐	☐	☐	☐	☐	☑
7	Blood Group	☐	☐	☐	☐	☐	☐	☑	☐	☐	☐
8	Pulse Rate (bpm)	☐	☐	☐	☐	☐	☐	☑	☐	☐	☐
9	Respiratory Rate (breaths/min)	☐	☐	☐	☑	☐	☐	☐	☐	☐	☐
10	Haemoglobin (Hb g/dl)	☐	☐	☐	☐	☐	☐	☐	☐	☐	☑
11	Cycle Regularity (R/I)	☐	☐	☐	☐	☐	☐	☑	☐	☐	☐
12	Cycle Length (days)	☐	☐	☐	☐	☐	☐	☑	☐	☐	☐
13	Marriage Status (yrs)	☐	☐	☐	☐	☐	☐	☑	☐	☐	☐
14	Pregnant (Y/N)	☐	☐	☐	☐	☐	☑	☐	☐	☐	☐
15	No. of Abortions	☐	☐	☐	☐	☐	☑	☐	☐	☐	☐
16	Beta-HCG I (mIU/mL)	☐	☐	☐	☐	☐	☐	☑	☐	☐	☐
17	Beta-HCG II (mIU/mL)	☐	☐	☐	☐	☐	☐	☑	☐	☐	☐
18	FSH (mIU/mL)	☐	☐	☐	☐	☐	☐	☑	☐	☐	☐
19	LH (mIU/mL)	☐	☐	☐	☐	☐	☐	☑	☐	☐	☐
20	FSH/LH Ratio	☐	☐	☐	☐	☐	☐	☑	☐	☐	☐
21	Hip (inch)	☐	☐	☐	☐	☐	☐	☑	☐	☐	☐

22	Waist (inch)	☐	☐	☐	☐	☐	☐	☐	☑	☐	☐
23	Waist-Hip Ratio	☐	☐	☐	☐	☐	☐	☐	☐	☐	☑
24	TSH (mIU/L)	☐	☐	☐	☐	☐	☐	☐	☐	☐	☑
25	AMH (ng/mL)	☐	☐	☐	☐	☐	☐	☐	☐	☐	☑
26	PRL (Prolactin ng/mL)	☐	☐	☐	☐	☐	☐	☐	☐	☐	☑
27	Vitamin D3 (ng/mL)	☐	☐	☐	☐	☐	☐	☐	☐	☐	☑
28	Progesterone (PRG ng/mL)	☐	☐	☐	☐	☐	☐	☐	☐	☐	☑
29	RBS (Random Blood Sugar mg/dl)	☐	☐	☐	☐	☐	☐	☐	☐	☐	☑
30	Weight Gain (Y/N)	☐	☐	☐	☐	☐	☐	☐	☐	☐	☑
31	Hair Growth (Y/N)	☐	☐	☐	☐	☐	☐	☐	☐	☐	☑
32	Skin Darkening (Y/N)	☐	☐	☐	☐	☐	☐	☐	☐	☐	☑
33	Hair Loss (Y/N)	☐	☐	☐	☐	☐	☐	☐	☐	☐	☑
34	Pimples (Y/N)	☐	☐	☐	☐	☐	☐	☐	☐	☐	☑
35	Fast Food Consumption (Y/N)	☐	☐	☐	☐	☐	☐	☑	☐	☐	☐
36	Regular Exercise (Y/N)	☐	☐	☐	☐	☐	☐	☐	☐	☐	☑
37	Systolic BP (mmHg)	☐	☐	☐	☐	☐	☐	☐	☐	☐	☑
38	Diastolic BP (mmHg)	☐	☐	☐	☐	☐	☐	☐	☐	☐	☑
39	Follicle Count (Left Ovary)	☐	☐	☐	☐	☐	☐	☐	☐	☐	☑
40	Follicle Count (Right Ovary)	☐	☐	☐	☐	☐	☐	☐	☐	☐	☑
41	Average Follicle Size (Left) (mm)	☐	☐	☐	☐	☐	☐	☐	☐	☐	☑
42	Average Follicle Size (Right) (mm)	☐	☐	☐	☐	☐	☐	☐	☐	☐	☑
43	Endometrium Thickness (mm)	☐	☐	☐	☐	☐	☐	☐	☑	☐	☐

Medical Officer
Sri Narayani Hospital and Research Cent--
VIT University Campus
Mobile No. 95664 20859

Dataset 2

Sl. No.	Feature	1	2	3	4	5	6	7	8	9	10
1	Country	☑	☐	☐	☐	☐	☐	☐	☐	☐	☐
2	Age	☐	☐	☐	☐	☐	☐	☐	☐	☐	☑
3	BMI	☐	☐	☐	☐	☐	☐	☐	☐	☐	☑
4	Menstrual Regularity	☐	☐	☐	☐	☐	☐	☐	☐	☐	☑
5	Hirsutism	☐	☐	☐	☐	☐	☐	☐	☐	☐	☑
6	Acne Severity	☐	☐	☐	☐	☐	☐	☐	☐	☐	☑
7	Family History of PCOS	☐	☐	☐	☐	☐	☐	☐	☐	☐	☑
8	Insulin Resistance	☐	☐	☐	☐	☐	☐	☐	☐	☐	☑
9	Lifestyle Score	☐	☐	☐	☐	☐	☐	☐	☐	☐	☑
10	Stress Levels	☐	☐	☐	☐	☐	☐	☐	☐	☐	☑
11	Urban/Rural	☐	☐	☐	☐	☐	☐	☐	☑	☐	☐
12	Socioeconomic Status	☐	☐	☐	☐	☐	☐	☐	☐	☑	☐
13	Awareness of PCOS	☐	☐	☐	☐	☐	☐	☐	☐	☑	☐
14	Fertility Concerns	☐	☐	☐	☐	☐	☐	☐	☐	☑	☐
15	Undiagnosed PCOS Likelihood	☐	☐	☐	☐	☐	☐	☐	☐	☑	☐
16	Ethnicity	☐	☐	☐	☐	☐	☐	☐	☐	☑	☐
17	Diagnosis	☐	☐	☐	☐	☐	☐	☐	☐	☑	☐

Medical Officer
Sri Narayani Hospital and Research Centre
VIT University Campus
Mobile No. 95664 20859

Request for Expert Opinion on PCOS Dataset Feature Weighting

From

D. Priyanka [23PHD0266],
Research Scholar,
SCORE, VIT, Vellore.
priyanka.d@vit.ac.in
9597036629

To

The Medical Officer,
Govt. GH,
Gudiyattam,

Subject: Seeking Expert Opinion on PCOS Risk Factors & Feature Weighting for Research.

Dear Dr. I. CATHERINE, MBBS.,DGO.,

I hope this letter finds you well. I am D. Priyanka, a research Scholar/PhD Student at VIT Vellore, working under the guidance of Dr.Sumangali.K, Associate Professor ,SCORE . I am currently conducting a research study on **Polycystic Ovary Syndrome (PCOS) prediction and diagnosis through machine learning and Deep Learning techniques.**

As part of this study, I aim to assign **relative importance (weights) to key clinical and biochemical features** related to PCOS, based on expert medical knowledge. Your insights as a specialist in [Endocrinology/Gynaecology/Related Field] would be invaluable in ensuring the accuracy and reliability of the feature selection process.

I have two datasets related to PCOS and prepared a table listing **43 features and 17 features**, including **BMI, hormonal levels, ultrasound findings, and lifestyle factors**. I kindly request your expert opinion on assigning **weights (1-10)** to each feature based on its significance in diagnosing or predicting PCOS.

Your feedback will play a crucial role in refining the feature selection process and improving the **clinical applicability of AI-based PCOS prediction models.** Please find the table attached for your review. I sincerely appreciate your time and expertise in supporting this research.

Looking forward to your valuable insights.

Thanking you,

Sincerely,

D. Priyanka [23PHD0266],
Research Scholar,
SCORE, VIT, Vellore.

Under the Guidance of

Dr. Sumangali .K,
Associate Professor,
SCORE, VIT, Vellore.

Dataset 1

Sl. No.	Feature	1 😐	2 😐	3 🙂	4 🙂	5 😋	6 😋	7 😃	8 😃	9 🤩	10 🤩
1	Patient File No.	☑	☐	☐	☐	☐	☐	☐	☐	☐	☐
2	PCOS (Y/N)	☐	☐	☐	☐	☐	☐	☐	☐	☐	☑
3	Age (yrs)	☐	☐	☐	☐	☐	☐	☐	☐	☐	☑
4	Weight (Kg)	☐	☐	☐	☐	☐	☐	☐	☐	☐	☑
5	Height (Cm)	☑	☐	☐	☐	☐	☐	☐	☐	☐	☐
6	BMI (Body Mass Index)	☐	☐	☐	☐	☐	☐	☐	☐	☐	☑
7	Blood Group	☑	☐	☐	☐	☐	☐	☐	☐	☐	☐
8	Pulse Rate (bpm)	☑	☐	☐	☐	☐	☐	☐	☐	☐	☐
9	Respiratory Rate (breaths/min)	☑	☐	☐	☐	☐	☐	☐	☐	☐	☐
10	Haemoglobin (Hb g/dl)	☑	☐	☐	☐	☐	☐	☐	☐	☐	☐
11	Cycle Regularity (R/I)	☐	☐	☐	☐	☐	☐	☐	☐	☐	☑
12	Cycle Length (days)	☐	☐	☐	☐	☐	☐	☐	☐	☐	☑
13	Marriage Status (yrs)	☐	☐	☐	☐	☐	☐	☐	☐	☐	☑
14	Pregnant (Y/N)	☑	☐	☐	☐	☐	☐	☐	☐	☐	☐
15	No. of Abortions	☐	☐	☐	☐	☐	☐	☐	☑	☐	☐
16	Beta-HCG I (mIU/mL)	☐	☐	☐	☐	☑	☐	☐	☐	☐	☐
17	Beta-HCG II (mIU/mL)	☐	☐	☐	☐	☑	☐	☐	☐	☐	☐
18	FSH (mIU/mL)	☐	☐	☐	☐	☐	☐	☐	☐	☐	☑
19	LH (mIU/mL)	☐	☐	☐	☐	☐	☐	☐	☐	☐	☑
20	FSH/LH Ratio	☐	☐	☐	☐	☐	☐	☐	☐	☐	☑
21	Hip (inch)	☐	☐	☐	☐	☐	☐	☐	☑	☐	☐

22	Waist (inch)	☐	☐	☐	☐	☐	☐	☐	☐	☐	☑
23	Waist-Hip Ratio	☐	☐	☐	☐	☐	☐	☐	☑	☐	☐
24	TSH (mIU/L)	☐	☐	☐	☐	☐	☐	☐	☐	☐	☑
25	AMH (ng/mL)	☐	☐	☐	☐	☐	☐	☐	☐	☐	☑
26	PRL (Prolactin ng/mL)	☐	☐	☐	☐	☐	☐	☐	☑	☐	☐
27	Vitamin D3 (ng/mL)	☐	☐	☐	☐	☑	☐	☐	☐	☐	☐
28	Progesterone (PRG ng/mL)	☐	☐	☐	☐	☐	☐	☐	☐	☐	☑
29	RBS (Random Blood Sugar mg/dl)	☐	☐	☐	☐	☐	☐	☐	☑	☐	☐
30	Weight Gain (Y/N)	☐	☐	☐	☐	☐	☐	☐	☐	☐	☑
31	Hair Growth (Y/N)	☐	☐	☐	☐	☐	☐	☐	☐	☐	☑
32	Skin Darkening (Y/N)	☐	☐	☐	☐	☐	☐	☐	☐	☐	☑
33	Hair Loss (Y/N)	☐	☐	☐	☐	☑	☐	☐	☐	☐	☐
34	Pimples (Y/N)	☐	☐	☐	☐	☐	☐	☐	☐	☐	☑
35	Fast Food Consumption (Y/N)	☐	☐	☐	☐	☑	☐	☐	☐	☐	☐
36	Regular Exercise (Y/N)	☐	☐	☐	☐	☐	☐	☐	☑	☐	☐
37	Systolic BP (mmHg)	☐	☐	☐	☐	☐	☑	☐	☐	☐	☐
38	Diastolic BP (mmHg)	☐	☐	☐	☐	☑	☐	☐	☐	☐	☐
39	Follicle Count (Left Ovary)	☐	☐	☐	☐	☐	☐	☐	☐	☐	☑
40	Follicle Count (Right Ovary)	☐	☐	☐	☐	☐	☐	☐	☐	☐	☑
41	Average Follicle Size (Left) (mm)	☐	☐	☐	☐	☑	☐	☐	☐	☐	☐
42	Average Follicle Size (Right) (mm)	☐	☐	☐	☐	☑	☐	☐	☐	☐	☐
43	Endometrium Thickness (mm)	☐	☐	☐	☐	☑	☐	☐	☐	☐	☐

Dr. I. CATHERINE, MBBS., DGO.,
TNMC Reg. No.55582
Sr. Civil Surgeon, Govt. GH
Gudiyattam, Vlr Dt.,

Dataset 2

Sl. No.	Feature	1 😐	2 😐	3 🙂	4 🙂	5 😊	6 😊	7 😃	8 😃	9 🤩	10 🤩
1	Country	☐	☐	☐	☐	☑	☐	☐	☐	☐	☐
2	Age	☐	☐	☐	☐	☐	☐	☐	☐	☐	☑
3	BMI	☐	☐	☐	☐	☐	☐	☐	☐	☐	☑
4	Menstrual Regularity	☐	☐	☐	☐	☐	☐	☐	☐	☐	☑
5	Hirsutism	☐	☐	☐	☐	☐	☐	☐	☐	☐	☑
6	Acne Severity	☐	☐	☐	☐	☐	☐	☐	☐	☐	☑
7	Family History of PCOS	☐	☐	☐	☐	☐	☐	☑	☐	☐	☐
8	Insulin Resistance	☐	☐	☐	☐	☐	☐	☐	☐	☐	☑
9	Lifestyle Score	☐	☐	☐	☐	☐	☐	☑	☐	☐	☐
10	Stress Levels	☐	☐	☐	☐	☐	☐	☑	☐	☐	☐
11	Urban/Rural	☐	☐	☐	☐	☐	☐	☐	☐	☐	☑
12	Socioeconomic Status	☐	☐	☐	☐	☐	☐	☐	☐	☐	☑
13	Awareness of PCOS	☐	☐	☐	☐	☐	☑	☐	☐	☐	☐
14	Fertility Concerns	☐	☐	☐	☐	☐	☐	☐	☐	☐	☑
15	Undiagnosed PCOS Likelihood	☐	☐	☐	☐	☐	☑	☐	☐	☐	☐
16	Ethnicity	☐	☐	☐	☐	☑	☐	☐	☐	☐	☐
17	Diagnosis	☐	☐	☐	☐	☐	☐	☐	☐	☐	☑

Reg NO 55581

Dr. I. CATHERINE, MBBS., DGO.,
TNMC Reg. No.55582
Sr. Civil Surgeon, Govt. GH
Gudiyattam, Vlr Dt.,

Request for Expert Opinion on PCOS Dataset Feature Weighting

From

D. Priyanka [23PHD0266],
Research Scholar,
SCORE, VIT, Vellore.
priyanka.d@vit.ac.in
9597036629

To

The Medical Officer,
MAA Hospital,
Katpadi.

Subject: Seeking Expert Opinion on PCOS Risk Factors & Feature Weighting for Research.

Dear Dr. K. Manjula, MBBS., M.S(OG),

I hope this letter finds you well. I am D. Priyanka, a research Scholar/PhD Student at VIT Vellore, working under the guidance of Dr.Sumangali.K, Associate Professor ,SCORE . I am currently conducting a research study on **Polycystic Ovary Syndrome (PCOS) prediction and diagnosis through machine learning and Deep Learning techniques.**

As part of this study, I aim to assign **relative importance (weights) to key clinical and biochemical features** related to PCOS, based on expert medical knowledge. Your insights as a specialist in [Endocrinology/Gynaecology/Related Field] would be invaluable in ensuring the accuracy and reliability of the feature selection process.

I have two datasets related to PCOS and prepared a table listing **43 features and 17 features**, including **BMI, hormonal levels, ultrasound findings, and lifestyle factors**. I kindly request your expert opinion on assigning **weights (1-10)** to each feature based on its significance in diagnosing or predicting PCOS.

Your feedback will play a crucial role in refining the feature selection process and improving the **clinical applicability of AI-based PCOS prediction models.** Please find the table attached for your review. I sincerely appreciate your time and expertise in supporting this research.

Looking forward to your valuable insights.

Thanking you,

Sincerely,

D. Priyanka [23PHD0266],
Research Scholar,
SCORE, VIT, Vellore.

Under the Guidance of

Dr. Sumangali .K,
Associate Professor,
SCORE, VIT, Vellore.

Dataset 1

Sl. No.	Feature	1	2	3	4	5	6	7	8	9 ☐	10 ☐
1	Patient File No.	☐	☐	☐	☐	☐	☐	☐	☐	☐	☑
2	PCOS (Y/N)	☐	☐	☐	☐	☐	☐	☐	☐	☐	☐
3	Age (yrs)	☐	☐	☐	☐	☐	☐	☐	☐	☐	☑
4	Weight (Kg)	☐	☐	☐	☐	☐	☐	☐	☐	☐	☑
5	Height (Cm)	☑	☐	☐	☐	☐	☐	☐	☐	☐	☑
6	BMI (Body Mass Index)	☐	☐	☐	☐	☐	☐	☐	☐	☐	☑
7	Blood Group	☑	☐	☐	☐	☐	☐	☐	☐	☐	☐
8	Pulse Rate (bpm)	☑	☐	☐	☐	☐	☐	☐	☐	☐	☐
9	Respiratory Rate (breaths/min)	☑	☐	☐	☐	☐	☐	☐	☐	☐	☐
10	Haemoglobin (Hb g/dl)	☑	☐	☐	☐	☐	☐	☐	☐	☐	☐
11	Cycle Regularity (R/I)	☐	☐	☐	☐	☐	☐	☐	☐	☐	☑
12	Cycle Length (days)	☐	☐	☐	☐	☐	☐	☐	☐	☐	☑
13	Marriage Status (yrs)	☐	☐	☐	☐	☐	☐	☐	☐	☐	☑
14	Pregnant (Y/N)	☐	☐	☐	☐	☐	☐	☐	☐	☐	☑
15	No. of Abortions	☐	☐	☐	☐	☐	☐	☐	☐	☐	☑
16	Beta-HCG I (mIU/mL)	☑	☐	☐	☐	☐	☐	☐	☐	☐	☐
17	Beta-HCG II (mIU/mL)	☑	☐	☐	☐	☐	☐	☐	☐	☐	☐
18	FSH (mIU/mL)	☐	☐	☐	☐	☐	☐	☐	☐	☐	☑
19	LH (mIU/mL)	☐	☐	☐	☐	☐	☐	☐	☐	☐	☑
20	FSH/LH Ratio	☐	☐	☐	☐	☐	☐	☐	☐	☐	☑
21	Hip (inch)	☐	☐	☐	☐	☐	☐	☐	☐	☐	☑

22	Waist (inch)	☐	☐	☐	☐	☐	☐	☐	☐	☐	☑
23	Waist-Hip Ratio	☐	☐	☐	☐	☐	☐	☐	☐	☐	☑
24	TSH (mIU/L)	☑	☐	☐	☐	☑	☐	☐	☐	☐	☐
25	AMH (ng/mL)	☐	☐	☐	☐	☑	☐	☐	☐	☐	☐
26	PRL (Prolactin ng/mL)	☐	☐	☑	☐	☐	☐	☐	☐	☐	☐
27	Vitamin D3 (ng/mL)	☑	☐	☐	☐	☐	☐	☐	☐	☐	☐
28	Progesterone (PRG ng/mL)	☐	☐	☐	☐	☑	☐	☐	☐	☐	☐
29	RBS (Random Blood Sugar mg/dl)	☐	☐	☐	☐	☐	☐	☐	☐	☐	☑
30	Weight Gain (Y/N)	☐	☐	☐	☐	☐	☐	☐	☐	☐	☑
31	Hair Growth (Y/N)	☐	☐	☐	☐	☐	☐	☐	☐	☐	☑
32	Skin Darkening (Y/N)	☐	☐	☐	☐	☐	☐	☐	☐	☐	☑
33	Hair Loss (Y/N)	☑	☐	☐	☐	☐	☐	☐	☐	☐	☐
34	Pimples (Y/N)	☐	☐	☐	☐	☐	☐	☐	☐	☐	☑
35	Fast Food Consumption (Y/N)	☐	☐	☐	☐	☑	☐	☐	☐	☐	☐
36	Regular Exercise (Y/N)	☐	☐	☐	☐	☐	☐	☐	☐	☐	☑
37	Systolic BP (mmHg)	☑	☐	☐	☐	☐	☐	☐	☐	☐	☐
38	Diastolic BP (mmHg)	☑	☐	☐	☐	☐	☐	☐	☐	☐	☐
39	Follicle Count (Left Ovary)	☑	☐	☐	☐	☐	☐	☐	☐	☐	☐
40	Follicle Count (Right Ovary)	☑	☐	☐	☐	☐	☐	☐	☐	☐	☐
41	Average Follicle Size (Left) (mm)	☑	☐	☐	☐	☐	☐	☐	☐	☐	☐
42	Average Follicle Size (Right) (mm)	☑	☐	☐	☐	☐	☐	☐	☐	☐	☐
43	Endometrium Thickness (mm)	☐	☐	☐	☐	☐	☐	☐	☐	☐	☑

Dr. K. MANJULA, MBBS., M.S(OG)
Consultant (Reg no: 96627)
MAA HOSPITAL
No. 50, Vallimalai Road, Katpadi,
Vellore - 632007

Dataset 2

Sl. No.	Feature	1 😐	2 😐	3 🙂	4 🙂	5 😊	6 😊	7 😁	8 😁	9 ☐	10 ☐
1	Country	☑	☐	☐	☑	☐	☐	☐	☐	☐	☐
2	Age	☐	☐	☐	☐	☑	☐	☐	☐	☐	☐
3	BMI	☐	☐	☐	☐	☐	☐	☐	☐	☐	☑
4	Menstrual Regularity	☐	☐	☐	☐	☐	☐	☐	☐	☐	☑
5	Hirsutism	☐	☐	☐	☐	☐	☐	☐	☐	☐	☑
6	Acne Severity	☐	☐	☐	☐	☐	☐	☐	☐	☐	☑
7	Family History of PCOS	☐	☐	☐	☐	☐	☐	☑	☐	☐	☐
8	Insulin Resistance	☐	☐	☐	☐	☐	☐	☐	☐	☐	☑
9	Lifestyle Score	☐	☐	☐	☐	☐	☐	☐	☐	☐	☑
10	Stress Levels	☐	☐	☐	☐	☐	☐	☑	☐	☐	☐
11	Urban/Rural	☐	☐	☐	☐	☑	☐	☐	☐	☐	☐
12	Socioeconomic Status	☐	☐	☐	☐	☑	☐	☐	☐	☐	☐
13	Awareness of PCOS	☐	☐	☐	☐	☑	☐	☐	☐	☐	☐
14	Fertility Concerns	☐	☐	☐	☐	☐	☐	☐	☐	☐	☑
15	Undiagnosed PCOS Likelihood	☑	☐	☐	☐	☐	☐	☐	☐	☐	☐
16	Ethnicity	☑	☐	☐	☐	☐	☐	☐	☐	☐	☐
17	Diagnosis	☐	☐	☐	☐	☐	☐	☐	☐	☐	☑

Dr. K. MANJULA, MBBS., M.S(OG)
Consultant (Reg no: 96627)
MAA HOSPITAL
No. 50, Vallimalai Road, Katpadi,
Vellore - 632007

/

Request for Expert Opinion on PCOS Dataset Feature Weighting

From

D. Priyanka [23PHD0266],
Research Scholar,
SCORE, VIT, Vellore.
priyanka.d@vit.ac.in
9597036629

To

The Medical Officer,
Good Shepherd Clinic,
Vellore.

Subject: Seeking Expert Opinion on PCOS Risk Factors & Feature Weighting for Research.

Dear Dr. R. MARY STELLA , MBBS.,M.D.(OG),

I hope this letter finds you well. I am D. Priyanka, a research Scholar/PhD Student at VIT Vellore, working under the guidance of Dr.Sumangali.K, Associate Professor ,SCORE . I am currently conducting a research study on **Polycystic Ovary Syndrome (PCOS) prediction and diagnosis through machine learning and Deep Learning techniques.**

As part of this study, I aim to assign **relative importance (weights) to key clinical and biochemical features** related to PCOS, based on expert medical knowledge. Your insights as a specialist in [Endocrinology/Gynaecology/Related Field] would be invaluable in ensuring the accuracy and reliability of the feature selection process.

I have two datasets related to PCOS and prepared a table listing **43 features and 17 features**, including **BMI, hormonal levels, ultrasound findings, and lifestyle factors**. I kindly request your expert opinion on assigning **weights (1-10)** to each feature based on its significance in diagnosing or predicting PCOS.

Your feedback will play a crucial role in refining the feature selection process and improving the **clinical applicability of AI-based PCOS prediction models.** Please find the table attached for your review. I sincerely appreciate your time and expertise in supporting this research.

Looking forward to your valuable insights.

Thanking you,

Sincerely,

D. Priyanka

D. Priyanka [23PHD0266],
Research Scholar,
SCORE, VIT, Vellore.

Under the Guidance of

Dr. Sumangali .K,
Associate Professor,
SCORE, VIT, Vellore.

Sumangali .K

Dataset 1

Sl. No.	Feature	1	2	3	4	5	6	7	8	9	10
1	Patient File No.	☑	☐	☐	☐	☐	☐	☐	☐	☐	☐
2	PCOS (Y/N)	☐	☐	☐	☐	☐	☐	☐	☐	☐	☑
3	Age (yrs)	☐	☐	☐	☐	☐	☐	☐	☑	☐	☐
4	Weight (Kg)	☐	☐	☐	☐	☐	☐	☐	☐	☐	☑
5	Height (Cm)	☐	☐	☐	☐	☑	☐	☐	☐	☐	☐
6	BMI (Body Mass Index)	☐	☐	☐	☐	☐	☐	☐	☐	☐	☑
7	Blood Group	☐	☐	☐	☐	☐	☐	☑	☐	☐	☐
8	Pulse Rate (bpm)	☐	☐	☐	☐	☑	☐	☐	☐	☐	☐
9	Respiratory Rate (breaths/min)	☐	☐	☐	☑	☐	☐	☐	☐	☐	☐
10	Haemoglobin (Hb g/dl)	☐	☐	☐	☐	☐	☐	☐	☑	☐	☐
11	Cycle Regularity (R/I)	☐	☐	☐	☐	☐	☐	☐	☐	☐	☑
12	Cycle Length (days)	☐	☐	☐	☐	☐	☐	☐	☐	☐	☑
13	Marriage Status (yrs)	☐	☐	☐	☐	☑	☐	☐	☐	☐	☐
14	Pregnant (Y/N)	☐	☐	☐	☐	☐	☐	☑	☐	☐	☐
15	No. of Abortions	☐	☐	☐	☐	☐	☐	☑	☐	☐	☐
16	Beta-HCG I (mIU/mL)	☐	☐	☐	☐	☐	☑	☐	☐	☐	☐
17	Beta-HCG II (mIU/mL)	☐	☐	☐	☐	☐	☐	☑	☐	☐	☐
18	FSH (mIU/mL)	☐	☐	☐	☐	☐	☐	☐	☐	☑	☐
19	LH (mIU/mL)	☐	☐	☐	☐	☐	☐	☐	☐	☑	☐
20	FSH/LH Ratio	☐	☐	☐	☐	☐	☐	☐	☑	☐	☐
21	Hip (inch)	☐	☐	☐	☐	☐	☐	☐	☐	☑	☐

22	Waist (inch)	☐	☐	☐	☐	☐	☐	☐	☐	☐	☑
23	Waist-Hip Ratio	☐	☐	☐	☐	☐	☐	☐	☐	☑	☐
24	TSH (mIU/L)	☐	☐	☐	☐	☑	☐	☐	☐	☐	☐
25	AMH (ng/mL)	☐	☐	☐	☐	☐	☐	☑	☐	☐	☐
26	PRL (Prolactin ng/mL)	☐	☐	☐	☐	☐	☑	☐	☐	☐	☐
27	Vitamin D3 (ng/mL)	☐	☐	☐	☐	☑	☐	☐	☐	☐	☐
28	Progesterone (PRG ng/mL)	☐	☐	☐	☐	☐	☐	☑	☐	☐	☐
29	RBS (Random Blood Sugar mg/dl)	☐	☐	☐	☐	☑	☐	☐	☐	☐	☐
30	Weight Gain (Y/N)	☐	☐	☐	☐	☐	☐	☑	☐	☐	☐
31	Hair Growth (Y/N)	☐	☐	☐	☐	☐	☐	☐	☑	☐	☐
32	Skin Darkening (Y/N)	☐	☐	☐	☐	☐	☐	☐	☐	☐	☑
33	Hair Loss (Y/N)	☐	☐	☐	☐	☐	☑	☐	☐	☐	☐
34	Pimples (Y/N)	☐	☐	☐	☐	☐	☐	☐	☐	☑	☐
35	Fast Food Consumption (Y/N)	☐	☐	☐	☐	☑	☐	☐	☐	☐	☐
36	Regular Exercise (Y/N)	☐	☐	☐	☐	☐	☐	☑	☐	☐	☐
37	Systolic BP (mmHg)	☐	☐	☐	☐	☑	☐	☐	☐	☐	☐
38	Diastolic BP (mmHg)	☐	☐	☐	☐	☑	☐	☐	☐	☐	☐
39	Follicle Count (Left Ovary)	☐	☐	☐	☐	☐	☐	☐	☑	☐	☐
40	Follicle Count (Right Ovary)	☐	☐	☐	☐	☐	☐	☐	☑	☐	☐
41	Average Follicle Size (Left) (mm)	☐	☐	☐	☐	☐	☑	☐	☐	☐	☐
42	Average Follicle Size (Right) (mm)	☐	☐	☐	☐	☐	☑	☐	☐	☐	☐
43	Endometrium Thickness (mm)	☐	☐	☐	☐	☐	☐	☐	☑	☐	☐

Dr. R. Mary Stella. M.B.B.S., M.D.(OG)
Reg. No. 94241
Consultant Obstetrician & Gynaecologist
Good Shepherd Clinic,
Katpadi, Vellore - 632 007.

Dataset 2

Sl. No.	Feature	1	2	3	4	5	6	7	8	9	10
1	Country	☐	☐	☑	☐	☐	☐	☐	☐	☐	☐
2	Age	☐	☐	☐	☐	☑	☐	☐	☐	☐	☐
3	BMI	☐	☐	☐	☐	☐	☐	☐	☐	☐	☑
4	Menstrual Regularity	☐	☐	☐	☐	☐	☐	☐	☐	☐	☑
5	Hirsutism	☐	☐	☐	☐	☐	☐	☐	☐	☐	☑
6	Acne Severity	☐	☐	☐	☐	☐	☐	☐	☐	☐	☑
7	Family History of PCOS	☐	☐	☐	☐	☐	☐	☐	☑	☐	☐
8	Insulin Resistance	☐	☐	☐	☐	☐	☐	☐	☐	☐	☑
9	Lifestyle Score	☐	☐	☐	☐	☑	☐	☐	☐	☐	☐
10	Stress Levels	☐	☐	☐	☐	☑	☐	☐	☐	☐	☐
11	Urban/Rural	☐	☐	☐	☐	☐	☐	☐	☐	☐	☑
12	Socioeconomic Status	☐	☐	☐	☐	☐	☐	☐	☑	☐	☐
13	Awareness of PCOS	☐	☐	☐	☐	☐	☑	☐	☐	☐	☐
14	Fertility Concerns	☐	☐	☐	☐	☐	☐	☐	☐	☐	☑
15	Undiagnosed PCOS Likelihood	☐	☐	☐	☐	☐	☐	☐	☑	☐	☐
16	Ethnicity	☐	☐	☐	☐	☐	☑	☐	☐	☐	☐
17	Diagnosis	☐	☐	☐	☐	☐	☐	☐	☐	☑	☐

Dr. R. Mary Stella. M.B.B.S., M.D.(OG)
Reg. No. 94241
Consultant Obstetrician & Gynaecologist
Good Shepherd Clinic,
Katpadi, Vellore - 632 007.
